# Using multimarker screening to identify biomarkers associated with cardiovascular death in patients with atrial fibrillation

**DOI:** 10.1093/cvr/cvab262

**Published:** 2021-08-06

**Authors:** Tymon Pol, Ziad Hijazi, Johan Lindbäck, Jonas Oldgren, John H Alexander, Stuart J Connolly, John W Eikelboom, Michael D Ezekowitz, Christopher B Granger, Renato D Lopes, Salim Yusuf, Agneta Siegbahn, Lars Wallentin

**Affiliations:** Department of Medical Sciences, Cardiology, Uppsala University, Uppsala Science Park, SE-752 37 Uppsala, Sweden; Uppsala Clinical Research Center, Uppsala University, Uppsala, Sweden; Department of Medical Sciences, Cardiology, Uppsala University, Uppsala Science Park, SE-752 37 Uppsala, Sweden; Uppsala Clinical Research Center, Uppsala University, Uppsala, Sweden; Uppsala Clinical Research Center, Uppsala University, Uppsala, Sweden; Department of Medical Sciences, Cardiology, Uppsala University, Uppsala Science Park, SE-752 37 Uppsala, Sweden; Uppsala Clinical Research Center, Uppsala University, Uppsala, Sweden; Duke Clinical Research Institute, Duke Health, Durham, NC, USA; Population Health Research Institute, Hamilton, Canada; Population Health Research Institute, Hamilton, Canada; Thomas Jefferson University, Philadelphia, PA, USA; Cardiovascular Medicine, Lankenau Institute for Medical Research, Wynnewood, PA, USA; Duke Clinical Research Institute, Duke Health, Durham, NC, USA; Duke Clinical Research Institute, Duke Health, Durham, NC, USA; Population Health Research Institute, Hamilton, Canada; Uppsala Clinical Research Center, Uppsala University, Uppsala, Sweden; Department of Medical Sciences, Clinical Chemistry, Uppsala University, Uppsala, Sweden; Department of Medical Sciences, Cardiology, Uppsala University, Uppsala Science Park, SE-752 37 Uppsala, Sweden; Uppsala Clinical Research Center, Uppsala University, Uppsala, Sweden

**Keywords:** Atrial fibrillation, Biomarkers, Cardiovascular death, Proteomics, Risk

## Abstract

**Aims:**

Atrial fibrillation (AF) is associated with higher mortality. Biomarkers may improve the understanding of key pathophysiologic processes in AF that lead to death. Using a new multiplex analytic technique, we explored the association between 268 biomarkers and cardiovascular (CV) death in anticoagulated patients with AF.

**Methods and results:**

A case–cohort design with 1.8- to 1.9-year follow-up. The identification cohort included 517 cases and 4057 randomly selected patients from ARISTOTLE. The validation cohort included 277 cases and 1042 randomly selected controls from RE-LY. Plasma collected at randomization was analysed with conventional immunoassays and the OLINK proximity extension assay panels: CVDII, CVDIII, and Inflammation. Association between biomarkers and CV death was evaluated using Random Survival Forest, Boruta, and adjusted Cox-regression analyses. The biomarkers most strongly and consistently associated with CV death were as follows (hazard ratio for inter-quartile comparison [95% CI]): N-terminal pro-B-type natriuretic peptide [NT-proBNP; 1.63 (1.37–1.93)], cardiac troponin T [cTnT-hs; 1.60 (1.35–1.88)], interleukin-6 [IL-6; 1.29 (1.13–1.47)], growth differentiation factor-15 [GDF-15; 1.30 (1.10–1.53)], fibroblast growth factor 23 [FGF-23; 1.21 (1.10–1.33)], urokinase receptor [uPAR; 1.38 (1.16–1.64)], trefoil factor 3 [TFF3; 1.27 (1.10–1.46)], tumour necrosis factor receptor 1 [TNFR1; 1.21 (1.01–1.45)], TNF-related apoptosis-inducing ligand receptor 2 [TRAILR2; 1.18 (1.04–1.34)], and cathepsin L1 [CTSL1; 1.22 (1.07–1.39)].

**Conclusion:**

In this comprehensive screening of 268 biomarkers in anticoagulated patients with AF, the underlying mechanisms most strongly associated with CV death were cardiorenal dysfunction (NT-proBNP, cTnT-hs, CTSL1, TFF3), oxidative stress (GDF-15), inflammation (IL-6, GDF-15), calcium balance, vascular and renal dysfunction (FGF-23), fibrinolysis (suPAR), and apoptosis (TNFR1, TRAILR2). These findings provide novel insights into pathophysiologic aspects associated with CV death in AF.

**ClinicalTrials.gov identifier:**

NCT00412984 and NCT00262600.

**See the editorial comment for this article ‘Utilizing biomarkers in atrial fibrillation: the pros and cons’, by Wern Yew Ding ***et al***., https://doi.org/10.1093/cvr/cvab283.**

## 1. Introduction

Atrial fibrillation (AF) is the most common persistent arrhythmia and is associated with a higher risk of a wide range of cardiovascular (CV) complications including a two-fold higher CV mortality.^1^ Even though stroke-related death can largely be prevented by the use of anticoagulation,^2^ the residual risk of death in AF remains high; specifically, 7.0% mortality was reported in the Apixaban for Reduction in Stroke and Other Thromboembolic Events in Atrial Fibrillation (ARISTOTLE) trial, and the majority of deaths were CV deaths.^3,4^

In recent years, new evidence has emerged of the potential role of biomarkers in predicting outcomes in patients with AF. Recently, biomarkers have been shown to improve the prediction of adverse outcomes in anticoagulated patients with AF.^5–7^ They may also facilitate the understanding of key pathophysiologic processes for complications in AF, and the identification of potential additional targets for treatment, not least in patients with remaining high mortality despite oral anticoagulation. Several biomarkers reflecting different pathophysiological functions have been shown to be powerful predictors of CV death in addition to clinical risk factors, for example myocardial damage and stress [cardiac troponin T (hs-TnT) and N-terminal pro-B-type natriuretic peptide (NT-proBNP)]^5,6^ and markers of inflammation and oxidative stress [interleukin 6 (IL-6) and growth differentiation factor-15 (GDF-15)].^7,8^

Recent advances in analytical methods have made it possible for simultaneous analysis of a vast number of proteins and could aid in both improving the understanding of the disease and identifying new clinically useful biomarkers. The proximity extension assay (PEA) technology, a new proteomics polymerase chain reaction (PCR)-based method, allows simultaneous analysis of the concentration of 92 proteins by making use of only 1 µl of blood plasma.^9^ This technique uses multiple unique oligonucleotide-labelled antibody pairs that bind to their respective target protein and can later be quantified by qPCR. In this way, multiple proteins can be analysed simultaneously with exceptionally high specificity, resulting in a time and sample-size effective method.

Using the PEA technology, the aim of this multimarker substudy was to comprehensively screen for biomarkers associated with CV death to improve the understanding of the processes that may be involved in CV death in anticoagulated patients with AF.

## 2. Methods

### 2.1 Patient population

This biomarker substudy consisted of patients from the ARISTOTLE trial and the Randomized Evaluation of Long-Term Anticoagulation Therapy (RE-LY) trial. The details of both trial designs and results have been published previously.^3,4^ Patients from the ARISTOTLE trial were included in the biomarker identification cohort, and patients from RE-LY comprised the validation cohort.

#### 2.1.1 Identification cohort

In the original ARISTOTLE trial, a total of 18 201 patients with AF and at least one CHADS_2_ risk factor for stroke or systemic embolism were enrolled. CV death was a secondary outcome. The identification cohort was derived from the ARISTOTLE trial biomarker cohort where all biomarker data were available (*N* = 14 757) using a 1:4 case-cohort methodology. The identification cohort thus consisted of 517 cases with CV death during follow-up and 4057 randomly selected patients for comparison. The median and maximal lengths of follow-up were 1.8 and 4.1 years, respectively.

#### 2.1.2 Validation cohort

In the RE-LY trial, 18 113 patients with AF were enrolled. CV death was among the secondary outcomes. The validation cohort was derived from the RE-LY biomarker cohort where all biomarker data were available *N* = 5533 using a 1:4 case-cohort design. The validation cohort thus consisted of 277 cases with CV death during follow-up and 1042 randomly selected patients. The median and maximal lengths of follow-up were 1.9 and 3.0 years, respectively.

### 2.2 Outcome definition and study design

This study was based on a case–cohort design in which all cases and a random sample from the full cohort were selected.

The primary outcome for this multimarker substudy was CV death. In both the ARISTOTLE and RE-LY trials, death was classified as either vascular or non-vascular. Vascular death included cardiac death (e.g. death from heart failure, sudden cardiac death/arrhythmia, cardiac rupture) and other vascular deaths (e.g. all-cause stroke, pulmonary embolus, death from aortic disease and from non-stroke-related haemorrhage). For this study, the primary outcome (CV death) was defined as vascular death excluding death from non-stroke-related haemorrhage. In both trials, cause of death was centrally adjudicated using standardized criteria. Both trials comply with the Declaration of Helsinki, and approval by the appropriate ethics committees was obtained at all sites and all patients provided written informed consent.

### 2.3 Biochemical analyses

Blood samples were obtained at randomization and stored in aliquots at −70°C.

For the proteomics analyses, the Proseek Multiplex PEA panels CVDII, CVDIII, and Inflammation were used (Olink Proteomics, Uppsala, Sweden) and performed at the Clinical Biomarkers Facility, Science for Life Laboratory, Uppsala University, Uppsala, Sweden. Within each panel, 92 biomarkers are measured simultaneously by the binding of paired single-strand oligonucleotide-labelled antibodies to the target protein. The subsequent formation of double-stranded DNA amplicons enables quantification by the Fluidigm BioMark™ HD real-time PCR platform.^10^ Values are given as Normalized Protein Expression (NPX) and are log2-transformed (Supplementary material online, *Table S1*). The PEA assays have shown high reproducibility and repeatability with low intra-assay, inter-assay, and inter-site variation.^10^ Prior validation studies have also showed that biomarkers analysed with the PEA technique have an adequate concordance with conventional immunoassays.^11^

Initial multiplex biomarker analysis was performed in the identification cohort using all three PEA panels. Together these PEA panels allowed for measurement of 276 pre-selected proteins associated with CV disease and inflammation. However, 10 biomarkers were analysed on more than one panel, resulting in duplicates and reducing the total number of analysed biomarkers. Therefore, 266 unique protein biomarkers were measured in total using PEA methodology in the identification cohort. Because of the comparatively low number of biomarkers with strong CV death association using the PEA inflammation chip in the initial analyses from the identification cohort and for the purpose of cost effectiveness, only the CVD II and III panel (and not the inflammation panel) was later used for biomarker analyses in the validation cohort. Thus, only 184 biomarkers were measured in the validation group.

The plasma levels of cTnT-hs and NT-proBNP were analysed with electrochemiluminescence immunoassays with the Cobas^®^ Analytics e601 (Roche Diagnostics). GDF-15 levels were determined with the Elecsys GDF-15 pre-commercial assay kit P03. High-sensitivity IL-6 was measured using ELISA (R&D Systems Inc., Minneapolis, MN, USA). Cystatin C was analysed with the ARCHITECT system ci8200 (Abbott Laboratories, Abbott Park, IL, USA) using the particle-enhanced turbidimetric immunoassay (PETIA) from Gentian (Moss, Norway), and all analyses were performed at the Uppsala Clinical Research Center (UCR) laboratory at Uppsala University, Uppsala Sweden, and detailed previously.^5–8^ Plasma creatinine was measured centrally, and estimated glomerular filtration rate (eGFR) was calculated using the Chronic Kidney Disease Epidemiology Collaboration (CKD-EPI) equation.

### 2.4 Statistical analyses

The pairwise association between PEA biomarkers and established conventional biomarkers was assessed by the Spearman correlation.

A Random Survival Forest algorithm^12^ was used to evaluate the simultaneous association between variables and CV death. The evaluation included levels of 263 PEA markers, four conventional markers (NT-proBNP, cTnT-hs, GDF-15, and IL-6), renal function, and 13 clinical characteristics [randomized treatment, age, gender, body mass index (BMI), smoking, hypertension, diabetes, haemoglobin, and previous myocardial infarction, stroke/transient ischaemic attack (TIA), peripheral artery disease, heart failure, and bleeding]. The total of biomarkers analysed with the Random Survival Forest algorithm was therefore 268 (five analysed by conventional assays including renal function + 266 biomarkers analysed with PEA, excluding three PEA biomarker duplicates that were analysed by conventional analyses). The number of trees was 5000, splits were done according to a maximally selected statistic criterion, and the variables were ranked according to their permutation variable importance. Subjects with all PEA markers missing were excluded. There were only a few partially missing values, and these were singly imputed using multivariate imputations by chained equations with the R add-on package mice.^13^ An identical approach was used in the RE-LY evaluation, with a total of 184 PEA markers.

A Boruta algorithm^14^ was used for feature selection. In short, repeated Random Survival Forests were run in which a permuted copy of each variable was added to the data. The permuted versions of the variables represent variables with the same distribution as the original variable but with no correlation with the outcome. Features were either rejected as not better, and were removed from subsequent forests, or confirmed better than these noise variables. The procedure continued until no more variables were undecided, or the maximum number of runs, set to 100, was reached. The remaining variables were labelled tentative.

Weighted Cox-regression analyses were performed including each of the established standard immunoassays (naturally log-transformed) and the PEA biomarkers, one at a time, assuming a linear association with the log hazard rate. According to the case-cohort design, the patients randomly selected were given a weight inversely proportional to the sampling probability, that is, 1/0.2946, and all cases were given a weight of 1.0. The Cox-regression analyses were performed in two steps, first (Cox model 1) adjusted for baseline characteristics (age, gender, BMI, smoking, hypertension, diabetes, prior myocardial infarction, prior stroke/TIA, peripheral artery disease, heart failure, and randomized treatment), and second (Cox model 2) further adjusting for renal function (cystatin C in ARISTOTLE and CKD-EPI in RE-LY) and established biomarkers (NT-proBNP and cTnT-hs). Results were presented as the relative hazard for an inter-quartile difference of each marker with corresponding 95% confidence intervals and *P*-values. Thus, the hazard ratio can be interpreted as the relative hazard comparing the two biomarker values defining the inner 50% of the distribution, that is the third vs. the first quartile. On the inflammation panel, 16 of the proteins had more than 80% of the measurements below the limit of detection, and these were not included in the Cox-regression models. Therefore, the total amount of biomarkers included in the Cox analyses was 255.

Due to the very large number of biomarkers evaluated and an adequate number of events, only biomarkers confirmed by the Boruta analysis and with significant association in the adjusted Cox-regression analysis (Cox model 2), in both the identification and validation cohorts, were considered to have confirmed association with the risk of CV death.

All analyses were done using the R environment for statistical computing, version 3.3.1^15^ using the ranger^16^ package.

## 3. Results

Baseline characteristics of the identification and validation cohorts are shown in *Table 1*. The random sample of controls was representative for the full study cohort (Supplementary material online, *Table S2*). Patients that died from a CV cause during follow-up were older, were more likely to have had a previous CV event including heart failure, and had higher levels of established CV biomarkers. The relative concentrations of all 266 PEA biomarkers are shown in Supplementary material online, *Tables S1A and B*.

**Table 1:** Baseline characteristics and concentration of established biomarkers

	Identification cohort		Validation cohort	
Baseline characteristics	Random sample	CV death	Random sample	CV death
	*N* = 4057	*N* = 517	*N* = 1042	*N* = 277
Age (years)	70 (72–76)	73 (65–69)	72 (67–77)	75 (67–80)
Females	1491 (36.8%)	148 (28.6%)	392 (37.7%)	99 (34.5%)
Body mass index	28.6 (25.4–32.7)	27.2 (24.1–31.4)	27.9 (25.0–31.2)	27.4 (23.8–31.2)
Current smoker	355 (8.8%)	60 (11.6%)	76 (7.3%)	23 (8.0%)
Hypertension	3550 (87.5%)	441 (85.3%)	826 (79.5%)	220 (76.7%)
Diabetes	1022 (25.2%)	130 (25.1%)	218 (21.0%)	82 (28.6%)
Prior myocardial infarction	511 (12.6%)	122 (23.6%)	169 (16.3%)	85 (29.6%)
Prior stroke/TIA	733 (18.1%)	123 (23.8%)	208 (20.0%)	54 (18.8%)
Peripheral arterial disease	190 (4.7%)	47 (9.1%)	39 (3.8%)	15 (5.2%)
Heart failure	1232 (30.4%)	258 (49.9%)	293 (28.2%)	152 (53.0%)
CRP	2.2 (1.0–4.6)	2.8 (1.2–6.3)	2.5 (1.2–5.5)	3.7 (1.6–8.1)
Cystatin C (mg/L)	1.0 (0.8–1.2)	1.1 (0.9–1.4)	1.0 (0.8–1.2)	1.2 (1.0–1.5)
GDF-15 (ng/L)	1356.0 (961.0–2038.0)	1780.0 (1292.0–2868.0)	1460.0 (1086.0–2127.0)	1997.0 (1438.5–3191.5)
eGFR, CKD-EPI (mL/min)	74.7 (57.6–96.0)	62.7 (45.2–84.7)	65.1 (54.3–74.2)	58.8 (48.4–69.7)
Haemoglobin (g/dL)	14.2 (13.1–15.2)	14.0 (12.9–15.2)	14.2 (13.1–15.3)	13.7 (12.7–15.0)
IL-6 (ng/L)	2.3 (1.5–3.9)	3.2 (2.1–6.0)	2.3 (1.5–3.8)	3.6 (2.1–5.8)
NT-proBNP (ng/L)	683.0 (362.0–1219.8)	1289.0 (709.0–2435.0)	800.0 (375.5–1436.0)	1568.0 (925.0–2805.0)
cTnT-hs (ng/L)	10.7 (7.4–16.2)	17.6 (12.0–27.0)	11.8 (7.6–18.9)	21.1 (14.2–33.6)

Continuous variables presented as median (Q1–Q3). Categorical variables presented as numbers (percentage).

CRP, C-reactive protein; cTnT-hs, cardiac troponin T measured with high-sensitivity assay; eGFR, estimated glomerular filtration rate; GDF-15, growth differentiation factor 15; IL-6, interleukin-6; NT-proBNP, N-terminal pro-B-type natriuretic peptide; TIA, transient ischaemic attack.

### 3.1 Random Survival Forest analyses

**Figure 1:**
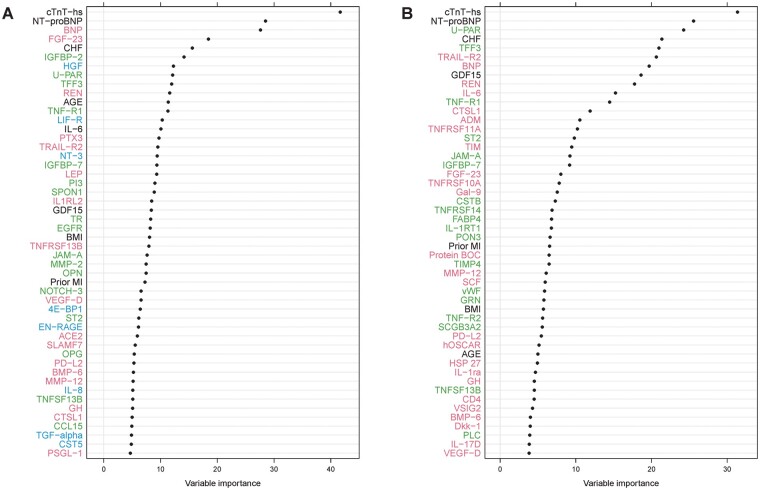
Variable importance for CV death according to the Random Survival Forest analyses in (*A*) the identification cohort and (*B*) the validation cohort. Red colour indicates biomarkers analysed on CVD II panel, green colour CVD III, and blue colour inflammation panel. Biomarkers listed in black were analysed with conventional immunoassays. Only the top 50 variables are shown. The evaluation included 263 PEA markers, five conventional markers [N-terminal pro-B-type natriuretic peptide (NT-proBNP), troponin T-hs, growth differentiation factor 15 (GDF-15), cystatin C, and interleukin-6 (IL-6)] and 13 clinical characteristics. The identification cohort included 517 cases with CV death during follow-up and 4057 randomly selected patients for comparison. The validation cohort included 277 cases with CV death during follow-up and 1042 randomly selected patients for comparison.

Among 268 studied biomarkers and 13 additional clinical risk factors examined in the identification cohort, the variables with the strongest association with CV death according to the Random Survival Forest analysis are shown in *Figure 1A*. The majority of the biomarkers most strongly associated with the outcome were from the PEA CVD II and CVD III panels, and only these panels were used for external validation while the PEA inflammation panel was excluded. In the validation cohort, out of 186 biomarkers and 12 clinical risk factors, the variables with strongest association with CV death are shown in *Figure 1B*.

In both cohorts, cTnT-hs was identified as having the strongest association with CV death according to the Random Survival Forest analysis and was followed by NT-proBNP. Due to the vast numbers of biomarkers consistently associated with the outcome, additional Boruta analyses were performed. According to the Boruta analysis, 32 biomarkers in the identification cohort (*Table 2*) and 29 biomarkers in the validation cohort (*Table 3*) had confirmed importance for CV death. In total, 15 biomarkers were consistently confirmed in both the identification and validation cohorts according to the Boruta analysis: cTnT-hs, NT-proBNP, BNP, fibroblast growth factor 23 (FGF-23), insulin-like growth factor-binding protein 2 (IGFBP2), fibrinolysis (suPAR), trefoil factor 3 (TFF3), renin (REN), tumour necrosis factor receptor 1 (TNFR1), IL-6, TNF-related apoptosis-inducing ligand receptor 2 (TRAILR2), insulin-like growth factor-binding protein 7 (IGFBP7), GDF-15, junctional adhesion molecule A (JAM-A), and cathepsin L1 (CTSL1).

**Table 2:** Summary of biomarkers with the highest association with cardiovascular death from the identification cohort

Biomarker	RF ranking	Model 1		Model 2		Boruta
		Hazard ratio (95% CI)	*P*-value	Hazard ratio (95% CI)	*P*-value	
**cTnT-hs**	**1**	**1.890 [1.630, 2.193]**	**<10e–16e**	**1.596 [1.355, 1.880]**	**2.174e–08**	**Confirmed**
**NT-proBNP**	**2**	**2.081 [1.772, 2.444]**	**<10e–16e**	**1.628 [1.373, 1.931]**	**2.179e–08**	**Confirmed**
BNP	3	2.016 [1.718, 2.365]	<10e**–**16e	1.361 [1.047, 1.769]	2.148e–02	Confirmed
**FGF-23**	**4**	**1.451 [1.341, 1.571]**	**<10e–16e**	**1.209 [1.096, 1.332]**	**1.408e–04**	**Confirmed**
IGFBP-2	5	1.788 [1.499, 2.133]	1.099e–10	1.288 [1.077, 1.540]	5.509e–03	Confirmed
HGF	6	1.268 [1.183, 1.358]	1.835e–11	1.085 [0.986, 1.194]	9.345e–02	Confirmed
**U-PAR**	**7**	**1.783 [1.550, 2.050]**	**5.551e–16**	**1.378 [1.158, 1.641]**	**3.105e–04**	**Confirmed**
**TFF3**	**8**	**1.528 [1.376, 1.697]**	**2.109e–15**	**1.269 [1.101, 1.462]**	**9.769e–04**	**Confirmed**
REN	9	1.168 [1.000, 1.366]	5.061e–02	1.152 [0.976, 1.360]	9.456e–02	Confirmed
**TNF-R1**	**10**	**1.598 [1.395, 1.831]**	**1.336e–11**	**1.207 [1.008, 1.447]**	**4.087e–02**	**Confirmed**
LIF-R	11	1.616 [1.410, 1.852]	5.666e–12	1.229 [1.055, 1.433]	8.316e–03	Confirmed
**IL-6**	**12**	**1.575 [1.410, 1.760]**	**9.992e–16**	**1.288 [1.126, 1.474]**	**2.338e–04**	**Confirmed**
PTX3	13	1.555 [1.357, 1.781]	2.031e–10	1.261 [1.091, 1.459]	1.733e–03	Confirmed
**TRAIL-R2**	**14**	**1.357 [1.249, 1.474]**	**4.613e–13**	**1.183 [1.043, 1.343]**	**9.037e–03**	**Confirmed**
NT-3	15	1.228 [1.123, 1.343]	6.769e–06	1.103 [0.993, 1.225]	6.652e–02	Confirmed
IGFBP-7	16	1.430 [1.284, 1.593]	8.415e–11	1.204 [1.068, 1.358]	2.487e–03	Confirmed
LEP	17	0.783 [0.652, 0.939]	8.545e–03	0.833 [0.686, 1.010]	6.275e–02	Confirmed
PI3	18	1.238 [1.110, 1.380]	1.219e–04	1.153 [1.024, 1.298]	1.904e–02	Confirmed
IL1RL2	20	0.764 [0.667, 0.876]	1.170e–04	0.794 [0.689, 0.914]	1.305e–03	Confirmed
**GDF-15**	**21**	**1.749 [1.532, 1.996]**	**1.110e–16**	**1.243 [1.050, 1.471]**	**1.142e–02**	**Confirmed**
TR	22	1.476 [1.308, 1.665]	2.637e–10	1.299 [1.141, 1.479]	8.092e–05	Confirmed
EGFR	23	0.831 [0.757, 0.912]	9.443e–05	0.861 [0.774, 0.959]	6.415e–03	Confirmed
TNFRSF13B	24	1.289 [1.176, 1.414]	6.732e–08	1.185 [1.064, 1.320]	1.949e–03	Confirmed
JAM-A	25	1.333 [1.210, 1.469]	5.954e–09	1.167 [1.031, 1.320]	1.423e–02	Confirmed
MMP-2	26	1.304 [1.133, 1.501]	2.211e–04	1.033 [0.904, 1.181]	6.340e–01	Confirmed
4E-BP1	30	1.331 [1.181, 1.499]	2.555e–06	1.202 [1.056, 1.369]	5.497e–03	Confirmed
EN-RAGE	32	1.267 [1.111, 1.444]	3.973e–04	1.147 [0.991, 1.327]	6.511e–02	Confirmed
ACE2	33	1.396 [1.234, 1.580]	1.211e–07	1.148 [1.000, 1.318]	5.018e–02	Confirmed
SLAMF1	34	1.237 [1.116, 1.372]	5.327e–05	1.112 [0.987, 1.251]	8.000e–02	Confirmed
TNFSF13B	40	1.257 [1.118, 1.412]	1.262e–04	1.097 [0.975, 1.235]	1.242e–01	Confirmed
**CTSL1**	**42**	**1.365 [1.207, 1.544]**	**7.328e–07**	**1.220 [1.069, 1.392]**	**3.173e–03**	**Confirmed**
TGFA	44	1.384 [1.214, 1.579]	1.210e–06	1.115 [0.983, 1.266]	9.156e–02	Confirmed

All biomarkers confirmed by Boruta analysis included, ranked according to Random Survival Forest (RF) variable importance. Biomarkers in **bold** indicate biomarkers *P* ≤ 0.05 in adjusted Cox-regression model 2 and confirmed in Boruta analyses in both cohorts. Model 1: Cox-regression analysis model adjusted for clinical characteristics—age, gender, body mass index, smoking, hypertension, diabetes, prior myocardial infarction, prior stroke/transient ischaemic attack, prior peripheral artery disease, prior heart failure, and randomized treatment. Model 2: same as Model 1 and also adjusted for marker for renal function, Cystatin C and cardiac markers N-terminal pro-B-type natriuretic peptide (NT-proBNP) + cardiac troponin T (hs-cTnT).

**Table 3:** Summary of biomarkers with the highest association with cardiovascular death from the validation cohort

Biomarker	RF ranking	Model 1		Model 2		Boruta
		Hazard ratio (95% CI)	*P*-value	Hazard ratio (95% CI)	*P*-value	
**cTnT-hs**	**1**	**1.753 [1.500–2.047]**	**1.464e–12**	**1.528 [1.305–1.788]**	**1.307e–07**	**Confirmed**
**NT-proBNP**	**2**	**2.959 [2.297–3.813]**	**<10e–16e**	**2.256 [1.758–2.894]**	**1.555e–10**	**Confirmed**
**U-PAR**	**3**	**2.193 [1.824–2.636]**	**1.110e–16**	**1.624 [1.300–2.028]**	**1.931e–05**	**Confirmed**
**TFF3**	**4**	**1.793 [1.492–2.154]**	**4.581e–10**	**1.518 [1.236–1.865]**	**7.028e–05**	**Confirmed**
**TRAIL-R2**	**5**	**1.343 [1.196–1.508]**	**6.012e–07**	**1.256 [1.121–1.407]**	**8.435e–05**	**Confirmed**
BNP	6	2.191 [1.673–2.870]	1.201e–08	1.073 [0.741–1.555]	0.70972	Confirmed
**GDF-15**	**7**	**1.980 [1.613–2.431]**	**6.749e–11**	**1.383 [1.100–1.739]**	**5.506e–03**	**Confirmed**
REN	8	1.825 [1.421–2.343]	2.385e–06	1.772 [1.380–2.274]	7.224e–06	Confirmed
**IL6**	**9**	**1.556 [1.379–1.757]**	**8.954e–13**	**1.310 [1.152–1.490]**	**3.729e–05**	**Confirmed**
**TNF-R1**	**10**	**2.153 [1.771–2.617]**	**1.377e–14**	**1.544 [1.207–1.974]**	**5.339e–04**	**Confirmed**
**CTSL1**	**11**	**1.684 [1.415–2.004]**	**4.241e–09**	**1.330 [1.107–1.599]**	**2.364e–03**	**Confirmed**
ADM	12	1.681 [1.133–2.494]	9.867e–03	1.092 [0.889–1.343]	0.40156	Confirmed
TNFRSF11A	13	2.062 [1.691–2.513]	7.775e–13	1.529 [1.210–1.931]	3.759e**–**04	Confirmed
ST2	14	1.622 [1.357–1.940]	1.127e–07	1.266 [1.056–1.517]	1.065e**–**02	Confirmed
TIM1	15	1.584 [1.317–1.904]	1.023e–06	1.317 [1.096–1.582]	3.317e**–**03	Confirmed
JAM-A	16	1.425 [1.245–1.632]	2.819e–07	1.168 [0.981–1.391]	8.162e**–**02	Confirmed
IGFBP-7	17	1.560 [1.361–1.788]	1.606e–10	1.178 [0.996–1.394]	5.635e**–**02	Confirmed
**FGF-23**	**18**	**1.476 [1.288–1.690]**	**1.907e–08**	**1.183 [1.023–1.368]**	**2.335e–02**	**Confirmed**
TNFRSF10A	19	1.844 [1.519–2.238]	6.347e–10	1.344 [1.078–1.677]	8.663e–03	Confirmed
GAL-9	20	1.966 [1.610–2.399]	3.012e–11	1.529 [1.212–1.930]	3.419e–04	Confirmed
CSTB	21	1.679 [1.432–1.968]	1.574e–10	1.357 [1.113–1.653]	2.474e–03	Confirmed
FABP4	23	2.159 [1.697–2.747]	3.749e–10	1.743 [1.347–2.254]	2.343e–05	Confirmed
IL-1RT1	24	1.521 [1.260–1.836]	1.286e–05	1.200 [0.999–1.441]	5.066e–02	Confirmed
PON3	25	0.684 [0.572–0.818]	3.159e–05	0.794 [0.651–0.969]	0.02328	Confirmed
BOC	26	1.470 [1.197–1.806]	2.372e–04	1.353 [1.123–1.631]	1.491e**–**03	Confirmed
MMP12	28	1.550 [1.274–1.886]	1.212e–05	1.322 [1.080–1.619]	6.845e**–**03	Confirmed
TNF-R2	32	1.415 [1.188–1.685]	9.948e–05	1.198 [1.009–1.422]	3.896e**–**02	Confirmed
CD4	40	1.799 [1.513–2.139]	2.923e–11	1.385 [1.125–1.704]	2.139e**–**03	Confirmed
IGFBP2	48	2.117 [1.621–2.764]	3.652e–08	1.290 [0.981–1.695]	6.796e–02	Confirmed

All biomarkers confirmed by Boruta analysis included, ranked according to Random Survival Forest (RF) variable importance. Biomarkers in **bold** indicate biomarkers *P* ≤ 0.05 in adjusted Cox-regression model 2 and confirmed in Boruta analyses in both cohorts. Model 1: Cox-regression analysis model adjusted for clinical characteristics—age, gender, body mass index, smoking, hypertension, diabetes, prior myocardial infarction, prior stroke/transient ischaemic attack, prior peripheral artery disease, prior heart failure, and randomized treatment. Model 2: same as Model 1 and also adjusted for marker for renal function, Cystatin C, and cardiac markers N-terminal pro-B-type natriuretic peptide (NT-proBNP) + cardiac troponin T (hs-cTnT).

### 3.2 Cox-regression analyses

**Figure 2:**
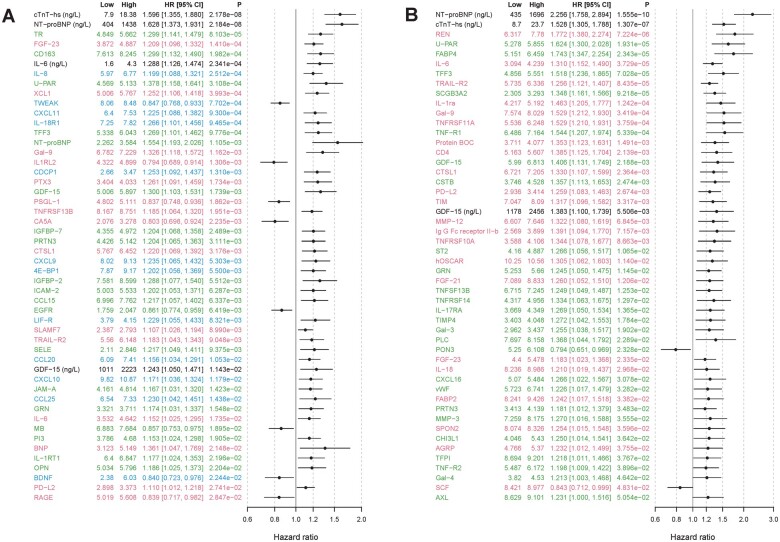
Forest plot of the top 50 biomarkers associated with CV death according to adjusted Cox-regression analysis in (*A*) the identification cohort and (*B*) the validation cohort (by *P*-value). A forest plot showing all 255 biomarkers is available in Supplementary material online, *Figures S1* and *S2*. Red colour indicates biomarkers analysed on CVD II panel, green colour CVD III, and blue colour inflammation panel. Biomarkers listed in black were analysed with conventional immunoassays. Model adjusted for baseline characteristics, renal function, and cardiac biomarkers [N-terminal pro-B-type natriuretic peptide (NT-proBNP), cardiac troponin T (cTnT-hs)]. The identification cohort included 517 cases with CV death during follow-up and 4057 randomly selected patients for comparison. The validation cohort included 277 cases with CV death during follow-up and 1042 randomly selected patients for comparison.

Evaluating the biomarker association of 255 biomarkers by Cox analyses adjusted solely for clinical characteristics (Cox model 1), 64% (*n* = 163) of the biomarkers were statistically associated with CV death in the identification cohort. In the validation cohort, the proportion was identical, and 64% (*n* = 121) out of 188 biomarkers were analysed. When further adjusted for renal function (Cystatin C) and for the two established biomarkers for CV death (NT-proBNP and cTnT-hs) (Cox model 2), 24% (*n* = 62) of the biomarkers in the identification cohort (top 50 shown in *Figure 2A*) and 26% (*n* = 49) in the validation cohort (top 50 shown in *Figure 2B*) remained significantly associated with CV death.

### 3.3 Biomarker selection

Out of the 15 biomarkers that were confirmed in the Boruta analysis in both the identification and validation cohorts, 10 were also determined statistically significant in the fully adjusted Cox analyses (model 2) in both cohorts: cTnT-hs, NT-proBNP, FGF-23, suPAR, TFF3, TNFR1, IL-6, TRAILR2, GDF-15, and CTSL1. A summary of the top candidate biomarkers according to the performed statistical analyses is presented in *Tables 2 and 3*. The correlation between these top biomarkers and traditional cardiovascular markers (NT-proBNP and cTnT-hs), marker for renal function (cystatin C) and for inflammation (CRP and IL-6), is shown in *Table 4*. suPAR, TFF3, TNFR1, IL-6, GDF-15, and TRAILR2 did all moderately correlate with renal function (rho >0.5). Beyond that, no strong patterns of correlation were seen (rho <0.5).

**Table 4:** Spearman correlation between the top candidate PEA biomarkers and established biomarkers

Biomarker	NT-proBNP	cTnT-hs	Cystatin C	Il-6	CRP
cTnT-hs	0.38	–	0.48	0.31	0.14
NT-proBNP	–	0.38	0.41	0.26	0.14
IL-6	0.26	0.31	0.28	–	0.52
GDF-15	0.35	0.50	0.52	0.36	0.17
suPAR	0.33	0.40	0.55	0.38	0.27
FGF-23	0.11	0.18	0.21	0.20	0.16
TRAILR2	0.39	0.49	0.64	0.37	0.20
TNFR1	0.29	0.45	0.64	0.33	0.24
TFF3	0.34	0.44	0.59	0.24	0.12
CTSL1	0.14	0.18	0.20	0.17	0.17

Correlation values presented from the identification cohort.

CRP, C-reactive protein; cTnT-hs, cardiac troponin T; CTSL1, cathepsin L1; FGF-23, fibroblast growth factor 23; GDF-15, growth differentiation factor 15; IL-6, interleukin-6; NT-proBNP, N-terminal pro-B-type natriuretic peptide; suPAR, urokinase receptor; TFF3, trefoil factor 3; TNFR1, tumour necrosis factor receptor 1; TRAILR2, TNF-related apoptosis-inducing ligand receptor 2.

## 4. Discussion

In this large biomarker substudy, we screened the prognostic importance of 268 protein biomarkers, measured by PEA multiplex and conventional immunoassays, on their association with CV death in two cohorts of anticoagulated patients with AF. Using a high statistical threshold with several modes of evaluations (Random Survival Forest/Boruta and adjusted Cox-regression analyses), 10 biomarkers were found to have a strong and consistent association with CV death in anticoagulated patients with AF. Of these, four were previously known: cTnT-hs, NT-proBNP, IL-6, and GDF-15, and six biomarkers novel in regard to their association with CV death in AF: FGF-23, suPAR, TFF3, TNFR1, TRAILR2, and CTSL1, and seem to reflect a spectrum of different pathological processes.

### 4.1 Biomarkers associated with CV death in AF

The two biomarkers having the strongest association with CV death in both trial cohorts were NT-proBNP and cTnT-hs. These cardiac biomarkers have in multiple studies shown to be independently associated with mortality in AF, as well as being strong risk predictors of death in a variety of cohorts and settings in patients without AF.^5,6,17^ In addition to death, these biomarkers are also associated with other CV outcomes in AF.^5,6^ These markers, reflecting myocardial stress and dysfunction, have also been shown to be associated with thromboembolic death.^6,18^ Our comprehensive study further emphasizes the superiority of NT-proBNP and cTnT-hs compared to all other 266 markers of inflammation and CV disease and 13 clinical risk factors including age. The findings strongly confirm the importance of these two biomarkers in regard to CV death in AF, even in the context of hundreds of additional biomarkers. However, in order to further expand our understanding of the mechanisms involved in CV death in AF, the evaluation of other biomarkers and disease processes is still important.

IL-6, an important mediator of inflammation with a causal role in heart disease,^19,20^ was among the top biomarkers for CV death in the present study. In AF, higher concentrations of IL-6 have been associated with higher AF burden and increased mortality.^8,21^ This study confirms previous findings of the importance of IL-6 regarding the risk of CV death in AF and IL-6 together with GDF-15 signify inflammation as a substantial pathophysiologic process in AF and a possible therapeutic target.^22^

GDF-15 is a marker of oxidative stress and inflammation and has in previous studies been shown to be a strong predictor of death in AF as well as other CV diseases.^7,23^ In our study, GDF-15 was confirmed as one of the biomarkers with the strongest association with CV mortality in AF. GDF-15 is upregulated by ageing, renal dysfunction, diabetes, CV diseases, inflammation, and is not specific for the heart. It is unclear precisely what role GDF-15 plays in AF. GDF-15 might work as a counter-regulatory factor by exerting cardioprotective effects in response to cardiovascular injury rather than causing the deleterious processes leading to death.^24^

Six other biomarkers less studied in AF, such as FGF-23, suPAR, TFF3, TNFR1, TRAILR2, and CTSL1, were identified as potential prognostic biomarkers with strong independent association with CV death in the present study. FGF-23 is a circulating peptide hormone that regulates phosphate and, indirectly, calcium balance.^25^ FGF-23 levels rise in chronic kidney disease and have been associated with cardiovascular events and mortality both in patients with ischaemic heart disease and in patients on haemodialysis, but also in a community-based cohort.^26–28^ Furthermore, in AF, FGF-23 has been shown to be associated with all-cause mortality in patients with end-stage renal disease,^29^ and several studies have also found an association between FGF-23 with incident AF.^30,31^ By displaying an association even after adjustment for cardio-renal markers, our findings extend upon these results and, for the first time, show a significant importance of FGF-23 for CV death in AF, suggesting that FGF-23 plays an important role in cardiovascular disease and in AF in particular. The exact mechanism of how FGF-23 is linked to CV death in AF is yet to be established but could include induction of left ventricular hypertrophy and cardiac remodelling,^32^ a mechanism that might in fact be reversible by therapeutic interventions.^33^ Further studies are needed to clarify the role of FGF-23 in cardiovascular disease and AF.

A novel finding was the independent association of suPAR with CV-death in AF. uPAR is an important component of the fibrinolytic system and is involved in cell migration and matrix degradation.^34^ During inflammatory stimulation, uPAR is cleaved from the cell surface of primarily immune cells resulting in a soluble form of uPAR which was the form analysed in this study. suPAR has been shown to be associated with cardiovascular disease, cancer and renal failure.^35,36^ In AF, suPAR has been explored as a predictor for incident AF however with diverging results.^37,38^ Our data indicate suPAR as having an important role in regards to mortality in AF—something that perhaps might be explained by the role of suPAR in the development of myocardial fibrosis and/or atherosclerosis, previously demonstrated in animal models.^39,40^

Another biomarker that was strongly associated with mortality in AF was TFF3, a member of the trefoil family. Data regarding the involvement of TFF3 in AF and heart disease are scarce but in experimental models TFF3 seems to be elevated during myocardial ischaemia, possibly enhancing ischaemic myocardial resistance.^40^ Whether TFF3 is an indirect marker of myocardial ischaemia or part of a novel pathophysiological process that leads to mortality in AF needs to be examined further.

TNFR1 is one of the receptors to which tumour necrosis factor alpha (TNF alpha) binds, mainly leading to necrosis or apoptosis.^41^ TNFR1 seems to be increased in heart failure patients compared to controls^42^ but does not, in our material, strongly correlate with NT-proBNP, another marker of heart failure. Evidence suggests that TNFR1 may participate in the pathophysiology of heart failure by mediating adverse remodelling.^43^ Furthermore, TNFR1 has been shown to be a predictor for incident heart failure, in particular for the heart failure with preserved ejection fraction (HFpEF) subtype, a condition that often co-exists with AF.^44^ The strong association between TNRF1 and mortality in AF has not, to our knowledge, been described previously. Our finding suggests the TNF alpha/TNFR1 system as a possible target for therapy for improving outcomes in patients with AF.

Similar to TNFR1, TRAILR2 is a marker of apoptosis and belongs to the tumour necrosis factor receptor superfamily.^45^ TRAILR2 concentrations do not seem affected by the presence of AF compared with sinus rhythm.^31^ TRAILR2 has previously been associated with heart failure and AF incidence and was also shown to predict mortality in patients after an acute myocardial infarction, perhaps reflecting inflammation and apoptotic activity.^31,46,47^ Our results extend the latter finding to the AF population suggesting that TRAIL-R2 is not specific for AF but rather a marker that rises in several disease states indicating poor prognosis.

CTSL1 is a lysosomal protease expressed in the heart and is involved in turnover and degradation of intra- and extracellular proteins.^48^ It is thought to help maintain normal cardiac function and morphology, something that was demonstrated by showing that CTSL1 knockout mice developed a dilated cardiomyopathy-like syndrome.^49^ Additionally, CTSL1 has in other animal models been shown to contribute to the repair and remodelling post-myocardial infarction,^50^ as well as exerting cardioprotective properties following pressure overload.^51^ There are limited data regarding CTSL1 in AF but the levels of CTSL1 in patients with AF in sinus rhythm are thought to be higher compared to patients without known history of AF.^52^ In the present study, CTSL1 emerged as an independent biomarker with strong association with CV death in AF. However, further research is needed to explore whether CTSL1 contributes to, or protects against, the deleterious processes leading to death from CV causes.

Many of these newly identified biomarkers with the highest association with CV death showed a correlation to renal function. However, in the present study, Cystatin C, an established renal function marker, did not show strong independent association with CV death in the Random Survival Forest analyses in comparison with the other biomarkers, nor after adjustment for NT-proBNP and cTnT-hs in the Cox analysis. This suggests that the association with CV death of these newly identified biomarkers was independent of renal function.

**Figure 3:**
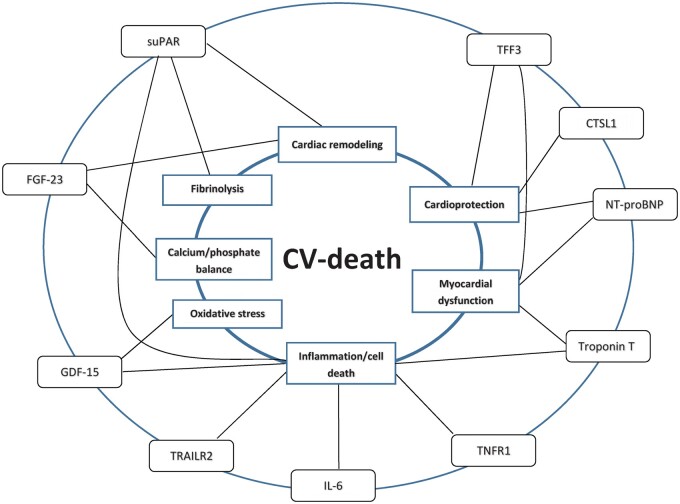
Conceptual figure showing top biomarkers and their associated processes in relation to CV death in AF.

In search for strategies to reduce the non-stroke-related mortality in patients with AF, exploring the underlying disease processes is of prime importance as understanding them better could facilitate the identification of patients at risk for death, thus allowing for earlier intervention, optimization of secondary prevention, and possibly even for targeted therapy to reduce AF-related mortality. This study identified several novel candidate biomarkers that reflect separate, although in many cases potentially overlapping biological pathways involved with CV death in AF, cardiac remodelling, cardio-renal dysfunction, inflammation, cell death, disturbances in calcium phosphate balance, fibrinolysis, and oxidative stress (*Figure 3*). The present study provides valuable insights into important processes involved with CV-death in patients with AF. Further studies are however needed for exploration of causal relationships and potential therapeutic interventions.

### 4.2 Strengths and limitations

The present study adds to current knowledge by using mass screening for identification of candidate biomarkers associated with CV death in AF, confirmed with validation in an independent dataset. The vast amount of proteins screened from two contemporary AF cohorts, the large number of events and sample size and the use of multiple statistical methods including both a linear and non-linear evaluation, is some of the strengths of this study.

Because of the exploratory nature of this study, adding multiplicity adjustment would unnecessarily increase the risk for type II error. The problem is somewhat alleviated by performing the screening in two separate study cohorts. Further, the individual Cox-regression analyses were combined with a random forest algorithm that handles all variables simultaneously and a Boruta algorithm to make inference about the significance of the variables’ importance. The Boruta algorithm as well as the Random Forest algorithm simultaneously handles all the variables and, thus, inherently also handles the multiplicity problem. Finally, since this is a screening study, the priority was in finding a set of top-ranked proteins, as found in both cohorts, and not so much in formal statistical significance.

The use of a conservative statistical approach applying two statistical methods for biomarker selection could result in an overly strict selection process and thereby fail to identify other potentially important biomarker candidates. However, the use of this approach adds robustness to the screening process and increases the certitude to the selection of candidate biomarkers. Also, the use of two biomarker PEA panels in the validation cohort, in contrast to three in the identification cohort, adds to the possibility of not identifying all biomarkers with strong association to CV death in AF.

The biomarker associations were studied in two AF populations but their specificity for the AF setting is not entirely clear.^53^ For example, GDF-15 is a predictive marker strongly associated with bleeding and death in AF, however it is also associated with poor outcomes outside the cardiovascular disease panorama.^54,55^ Because of the exploratory nature of this study, it is hard to draw any conclusions whether the biomarkers in this study solely reflect biological mechanisms linked to CV death in AF or more broadly, cardiovascular disease status, comorbidity burden, or even ageing.^56^ Further studies comparing AF populations with healthy controls and/or non-AF disease groups are necessary to study the specificity of the biomarkers and mechanisms for the AF setting.

The study population was anticoagulated, and our results may thus not be entirely generalizable to other populations. Another limitation is the lack of data regarding biomarker level change over time that could point out mechanisms involved in the processes leading up to death. Even though the statistical analyses adjusted for patient characteristics, cardiovascular risk factors, and biomarkers, residual confounding cannot be excluded.

Broad biomarker screening as performed in this study serves as a first step to identify pathophysiological processes of interest. It allows future studies to use more focussed mechanistic investigations and evaluate the identified biomarkers for risk prediction which in extension allows for the development of decision support tools to improve outcomes in the studied disease. While appropriate for protein screening purposes, the PEA analytic method provides relative protein concentrations only and thus, in further evaluation of clinical usefulness, quantitative assays should be preferred.

## 5. Conclusion

This comprehensive biomarker screening study to identify biomarkers associated with CV death in AF confirmed NT-proBNP, cTnT-hs, IL-6, and GDF-15 from previous studies and identified six additional novel biomarkers such as FGF-23, suPAR, TFF3, TNFR1, TRAILR2, and CTSL1, as the most important out of 268 biomarkers from two large cohorts. These findings provide valuable insights into important pathophysiologic processes that may be involved with cardiovascular death in patients with AF and that, in the future, might be modifiable.

## Supplementary material

Supplementary material is available at *Cardiovascular Research* online.

## Author’s contributions

T.P., Z.H., J.O., A.S., L.W., and J.L. are responsible for the conceptualization and formal analysis of the study results, and contributed to the writing and revision of the paper. T.P. wrote the original draft. All other authors contributed to the revision of the paper.

**Conflict of interest:** T.P.: none. Z.H.: lecture fees from Boehringer Ingelheim, Roche, Bristol-Myers Squibb, and Pfizer; consulting fees from Merck Sharp & Dohme, Roche, Bristol-Myers Squibb, and Pfizer. Research grants from the Swedish Society for Medical Research [S17-0133] and the Swedish Heart-Lung Foundation [20170718]. J.L.: Institutional research grants from Boehringer Ingelheim, Bristol-Myers Squibb/Pfizer. J.O.: fees to his institution from AstraZeneca, Bayer HealthCare, Boehringer Ingelheim, Bristol-Myers Squibb, Daiichi Sankyo, Pfizer, Portola, Roche Diagnostics, Sanofi. J.H.A.: institutional research grants from Bayer, Boehringer Ingelheim, Bristol-Myers Squibb, Cryolife, CSL Behring, Ferring, Glaxosmithkline, XaTek; consulting fees/honoraria from AbbVie, Bristol-Myers Squibb, Cryolife, Glaxosmithkline, Pfizer, Portola. S.J.C.: consulting fees, speaker fees, and research grants from Boehringer Ingelheim, Bristol‐Myers Squibb, Bayer, Portola; consulting fees and research grants from Sanofi‐Aventis; research grants from Boston Scientific. J.W.E.: Institutional research grants and honoraria from AstraZeneca, Bayer, Boehringer Ingelheim, Bristol‐Myers Squibb/Pfizer, Daiichi‐Sankyo, Eli Lilly, Glaxo Smith Kline, Janssen, Sanofi. M.D.E.: grants and consultant fees from Boehringer Ingelheim, Bristol Myers‐Squibb, Pfizer; consultant fees from Boston Scientific, Anthos Therapeutic, Alta Therapeutics. C.B.G.: grants and personal fees from GlaxoSmithKline, Boehringer Ingelheim, Bristol-Myers Squibb, Pfizer, Sanofi-Aventis, Takeda, The Medicines Company, Janssen, Bayer, Hoffmann-La Roche; grants from Medtronics Foundation, Merck & Co., Armetheon; personal fees from Lilly, AstraZeneca, Daiichi Sankyo, Ross Medical Corporation, Salix Pharmaceuticals, Gilead. R.D.L.: institutional research grant and consulting fees from Bristol-Myers Squibb; institutional research grant from GlaxoSmithKline; consulting fees from Bayer, Boehringer Ingleheim, Pfizer, Merck, Portola. S.Y.: grants, speaker fees, and paid travel expenses from Boehringer Ingelheim. A.S.: institutional research grants from AstraZeneca, Boehringer Ingelheim, Bristol-Myers Squibb/Pfizer, GlaxoSmithKline, Roche Diagnostics; consultancy fees from Olink Proteomics. L.W.: institutional research grants, consultancy fees, lecture fees, and travel support from Bristol-Myers Squibb/Pfizer, AstraZeneca, GlaxoSmithKline, Boehringer Ingelheim; institutional research grants from Merck & Co, Roche; consultancy fees from Abbott; holds two patents involving GDF-15.

## Funding

This work was supported by The Swedish Foundation for Strategic Research [grant number RB13-0197]; the Swedish Heart-Lung Foundation [grant number 20090183]; and Science for Life Laboratory, Uppsala University, Uppsala, Sweden. The ARISTOTLE trial was funded by Bristol-Myers Squibb Co, Princeton, NJ, USA and Pfizer Inc., New York, NY, USA, and coordinated by the Duke Clinical Research Institute, Durham, NC, USA and Uppsala Clinical Research Center, Uppsala, Sweden. The RE-LY trial was funded by Boehringer Ingelheim, Ingelheim, Germany. Roche Diagnostics, Rotkreuz, Switzerland, provided the pre-commercial assay of GDF-15. Roche Diagnostics was given the opportunity to review and comment on the final version of the manuscript.

## Data availability

The data, analytical methods, and study materials will not be made available to other researchers for purposes of reproducing the results or replicating the procedure.

Translational perspective

In patients with AF, there is an unmet need for better understanding of the pathophysiological processes involved with CV death. Using a targeted proteomic approach, 10 biomarkers were identified as having a strong association with CV death. The identified biomarkers reflect several biological pathways involved with CV death in AF. The present study provides valuable insights into important processes involved with CV death in patients with AF and may facilitate the identification of important risk factors for death, thus allowing for earlier intervention and possibly even for targeted therapy to reduce AF-related mortality.

## Supplementary Material

cvab262_Supplementary_Data

## Notes

**Time for primary review: 37 days.**
