# Supplementary material for: Using multimarker screening to identify biomarkers associated with cardiovascular death in patients with atrial fibrillation
Source: Cardiovasc Res. 2021 Aug 6;118(9):2112–23. doi: 10.1093/cvr/cvab262 (PMC9302885; doi:10.1093/cvr/cvab262)
Supplement: cvab262_Supplementary_Data [file cvab262_supplementary_data.docx]

**SUPPLEMENTAL APPENDIX**

**Using multimarker screening to identify biomarkers associated with cardiovascular death in patients with atrial fibrillation**

Tymon Pol, Ziad Hijazi, Johan Lindbäck, Jonas Oldgren, John H. Alexander, Stuart J. Connolly, John W. Eikelboom, Michael D. Ezekowitz, Christopher B. Granger, Renato D. Lopes M.D, Salim Yusuf, Agneta Siegbahn, and Lars Wallentin.

Contents

[Supplemental Table 1A 2](#_Toc58951030)

[Supplemental Table 1B 12](#_Toc58951031)

[Supplemental Table 2 19](#_Toc58951032)

# **Supplemental Table 1A**

Baseline levels of biomarkers expressed in NPX values (arbitrary units) for cases and controls in the identification cohort

| Variable |  | UniProt No. | No | CV death |
| --- | --- | --- | --- | --- |
| BMP-6 | Bone morphogenetic protein 6 | P22004 | 5.7 (5.4 -- 6.0) [197] | 5.8 (5.5 -- 6.2) [27] |
| ANG-1 | Angiopoietin-1 | Q15389 | 9.0 (8.1 -- 9.6) [197] | 8.8 (7.9 -- 9.6) [27] |
| ADM | ADM | P35318 | 7.5 (7.2 -- 7.8) [197] | 7.7 (7.4 -- 8.0) [27] |
| CD40-L | CD40 ligand | P29965 | 4.1 (3.5 -- 5.1) [197] | 4.2 (3.6 -- 5.3) [27] |
| SLAMF7 | SLAM family member 7 | Q9NQ25 | 2.4 (2.4 -- 2.7) [197] | 2.6 (2.4 -- 3.0) [27] |
| PlGF | Placenta growth factor | P49763 | 8.1 (7.9 -- 8.3) [197] | 8.2 (8.0 -- 8.5) [27] |
| ADAM-TS13 | A disintegrin and metalloproteinase with thrombospondin motifs 13 | Q76LX8 | 5.2 (5.1 -- 5.3) [197] | 5.2 (5.1 -- 5.3) [27] |
| Protein BOC | Brother of CDO | Q9BWV1 | 4.9 (4.7 -- 5.0) [197] | 4.9 (4.7 -- 5.1) [27] |
| IL-4RA | Interleukin-4 receptor subunit alpha | P24394 | 3.2 (2.9 -- 3.5) [197] | 3.3 (3.0 -- 3.7) [27] |
| SRC | Proto-oncogene tyrosine-protein kinase Src | P12931 | 5.5 (4.4 -- 6.9) [197] | 5.9 (4.5 -- 7.0) [27] |
| IL-1ra | Interleukin-1 receptor antagonist protein | P18510 | 4.5 (4.1 -- 5.0) [197] | 4.6 (4.1 -- 5.1) [27] |
| IL-6 | Interleukin-6 | P05231 | 4.0 (3.5 -- 4.5) [197] | 4.3 (3.7 -- 5.0) [27] |
| TNFRSF10A | Tumor necrosis factor receptor superfamily member 10A | O00220 | 3.7 (3.4 -- 3.9) [197] | 3.8 (3.5 -- 4.0) [27] |
| STK4 | Serine/threonine-protein kinase 4 | Q13043 | 1.9 (1.2 -- 3.5) [197] | 2.2 (1.2 -- 3.6) [27] |
| IDUA | Alpha-L-iduronidase | P35475 | 4.6 (4.1 -- 5.0) [197] | 4.5 (4.1 -- 4.9) [27] |
| TNFRSF11A | Tumor necrosis factor receptor superfamily member 11A | Q9Y6Q6 | 5.8 (5.5 -- 6.2) [197] | 6.0 (5.6 -- 6.4) [27] |
| PAR-1 | Proteinase-activated receptor 1 | P25116 | 7.4 (7.1 -- 7.7) [197] | 7.6 (7.2 -- 7.9) [27] |
| TRAIL-R2 | TNF-related apoptosis-inducing ligand receptor 2 | O14763 | 5.8 (5.5 -- 6.1) [197] | 6.0 (5.7 -- 6.3) [27] |
| PRSS27 | Serine protease 27 | Q9BQR3 | 8.1 (7.8 -- 8.4) [197] | 8.2 (7.9 -- 8.5) [27] |
| TIE2 | Angiopoietin-1 receptor | Q02763 | 8.1 (7.9 -- 8.2) [197] | 8.2 (8.0 -- 8.3) [27] |
| TF | Tissue factor | P13726 | 5.8 (5.6 -- 6.0) [197] | 5.9 (5.6 -- 6.1) [27] |
| IL1RL2 | Interleukin-1 receptor-like 2 | Q9HB29 | 4.6 (4.3 -- 4.9) [197] | 4.5 (4.2 -- 4.9) [27] |
| PDGF subunit B | Platelet-derived growth factor subunit B | P01127 | 8.9 (8.1 -- 9.6) [197] | 8.8 (7.9 -- 9.5) [27] |
| IL-27 | Interleukin-27 | Q8NEV9, Q14213 | 4.5 (4.2 -- 4.7) [197] | 4.6 (4.4 -- 4.8) [27] |
| IL-17D | Interleukin-17D | Q8TAD2 | 2.7 (2.6 -- 2.9) [197] | 2.8 (2.6 -- 3.0) [27] |
| CXCL1 | C-X-C motif chemokine 1 | P09341 | 8.3 (7.4 -- 8.9) [197] | 8.2 (7.4 -- 8.9) [27] |
| LOX-1 | Lectin-like oxidized LDL receptor 1 | P78380 | 6.9 (6.5 -- 7.3) [197] | 7.0 (6.7 -- 7.4) [27] |
| Gal-9 | Galectin-9 | O00182 | 7.0 (6.7 -- 7.2) [197] | 7.1 (6.9 -- 7.4) [27] |
| GIF | Gastric intrinsic factor | P27352 | 5.6 (5.0 -- 6.2) [197] | 5.6 (4.9 -- 6.3) [27] |
| SCF | Stem cell factor | P21583 | 9.7 (9.4 -- 9.9) [197] | 9.6 (9.3 -- 9.9) [27] |
| IL-18 | Interleukin-18 | Q14116 | 8.7 (8.4 -- 9.1) [197] | 8.8 (8.4 -- 9.2) [27] |
| FGF-21 | Fibroblast growth factor 21 | Q9NSA1 | 7.7 (6.8 -- 8.6) [197] | 7.9 (7.1 -- 9.1) [27] |
| PIgR | Polymeric immunoglobulin receptor | P01833 | 6.5 (6.4 -- 6.5) [197] | 6.5 (6.4 -- 6.6) [27] |
| RAGE | Receptor for advanced glycosylation end products | Q15109 | 5.3 (5.0 -- 5.6) [197] | 5.4 (5.0 -- 5.7) [27] |
| SOD2 | Superoxide dismutase [Mn], mitochondrial | P04179 | 8.9 (8.7 -- 9.0) [197] | 8.8 (8.7 -- 9.0) [27] |
| CTRC | Chymotrypsin C | Q99895 | 10.2 (9.7 -- 10.7) [197] | 10.2 (9.7 -- 10.7) [27] |
| FGF-23 | Fibroblast growth factor 23 | Q9GZV9 | 4.2 (3.8 -- 4.8) [197] | 4.6 (4.0 -- 5.6) [27] |
| SPON2 | Spondin-2 | Q9BUD6 | 9.0 (8.9 -- 9.1) [247] | 9.1 (8.9 -- 9.2) [39] |
| GH | Growth hormone | P01241 | 8.4 (7.0 -- 10.0) [197] | 9.5 (7.8 -- 10.7) [27] |
| FS | Follistatin | P19883 | 12.0 (11.7 -- 12.3) [197] | 12.0 (11.7 -- 12.3) [27] |
| GLO1 | Lactoylglutathione lyase | Q04760 | 6.1 (5.7 -- 6.7) [197] | 6.2 (5.8 -- 6.8) [27] |
| CD84 | SLAM family member 5 | Q9UIB8 | 5.3 (5.1 -- 5.6) [197] | 5.4 (5.1 -- 5.7) [27] |
| PAPPA | Pappalysin-1 | Q13219 | 3.2 (2.9 -- 3.6) [197] | 3.3 (3.0 -- 3.7) [27] |
| SERPINA12 | Serpin A12 | Q8IW75 | 3.6 (3.0 -- 4.4) [197] | 3.7 (3.0 -- 4.4) [27] |
| REN | Renin | P00797 | 7.5 (6.8 -- 8.1) [197] | 7.6 (6.8 -- 8.5) [27] |
| DECR1 | 2,4-dienoyl-CoA reductase, mitochondrial | Q16698 | 3.1 (2.5 -- 3.8) [197] | 3.1 (2.5 -- 3.9) [27] |
| MERTK | Tyrosine-protein kinase Mer | Q12866 | 4.5 (4.2 -- 4.7) [197] | 4.5 (4.3 -- 4.8) [27] |
| TIM | T-cell immunoglobulin mucin receptor 1 | Q96D42 | 10.0 (9.5 -- 10.6) [197] | 10.3 (9.6 -- 10.9) [27] |
| THBS2 | Thrombospondin-2 | P35442 | 6.0 (5.9 -- 6.2) [264] | 6.1 (5.9 -- 6.3) [36] |
| TM | Thrombomodulin | P07204 | 8.4 (8.2 -- 8.7) [197] | 8.5 (8.3 -- 8.8) [27] |
| VSIG2 | V-set and immunoglobulin domain-containing protein 2 | Q96IQ7 | 4.0 (3.6 -- 4.3) [197] | 4.1 (3.8 -- 4.6) [27] |
| AMBP | Protein AMBP | P02760 | 7.1 (7.0 -- 7.2) [197] | 7.1 (7.0 -- 7.3) [27] |
| PRELP | Prolargin | P51888 | 6.7 (6.6 -- 6.8) [197] | 6.8 (6.6 -- 6.9) [27] |
| HO-1 | Heme oxygenase 1 | P09601 | 11.7 (11.4 -- 11.9) [197] | 11.6 (11.4 -- 11.9) [27] |
| XCL1 | Lymphotactin | P47992 | 5.4 (5.0 -- 5.7) [197] | 5.6 (5.2 -- 5.9) [27] |
| IL16 | Pro-interleukin-16 | Q14005 | 5.3 (5.0 -- 5.6) [197] | 5.3 (5.0 -- 5.7) [27] |
| SORT1 | Sortilin | Q99523 | 6.4 (6.2 -- 6.5) [197] | 6.4 (6.2 -- 6.6) [27] |
| CEACAM8 | Carcinoembryonic antigenrelated cell adhesion molecule 8 | P31997 | 4.2 (3.9 -- 4.7) [197] | 4.4 (4.0 -- 4.8) [27] |
| PTX3 | Pentraxin-related protein PTX3 | P26022 | 3.7 (3.4 -- 4.0) [197] | 3.9 (3.5 -- 4.2) [27] |
| PSGL-1 | P-selectin glycoprotein ligand 1 | Q14242 | 5.0 (4.8 -- 5.1) [197] | 4.9 (4.7 -- 5.1) [27] |
| CCL17 | C-C motif chemokine 17 | Q92583 | 6.9 (6.3 -- 7.6) [197] | 6.8 (6.2 -- 7.7) [27] |
| CCL3 | C-C motif chemokine 3 | P10147 | 2.8 (2.5 -- 3.1) [197] | 3.0 (2.7 -- 3.3) [27] |
| MMP-7 | Matrix metalloproteinase-7 | P09237 | 7.3 (6.3 -- 8.1) [197] | 7.4 (6.3 -- 8.3) [27] |
| Ig G Fc receptor II-b | Low affinity immunoglobulin gamma Fc region receptor II-b | P31994 | 1.7 (1.4 -- 2.1) [197] | 1.8 (1.5 -- 2.1) [27] |
| ITGB1BP2 | Melusin | Q9UKP3 | 3.0 (3.0 -- 4.5) [197] | 3.3 (3.0 -- 4.8) [27] |
| DCN | Decorin | P07585 | 5.4 (5.3 -- 5.6) [197] | 5.5 (5.3 -- 5.7) [27] |
| Dkk-1 | Dickkopf-related protein 1 | O94907 | 8.9 (8.5 -- 9.3) [197] | 8.9 (8.5 -- 9.3) [27] |
| LPL | Lipoprotein lipase | P06858 | 9.5 (9.2 -- 9.8) [197] | 9.6 (9.3 -- 9.8) [27] |
| PRSS8 | Prostasin | Q16651 | 9.2 (9.0 -- 9.5) [197] | 9.3 (9.0 -- 9.5) [27] |
| AGRP | Agouti-related protein | O00253 | 3.2 (3.0 -- 3.5) [197] | 3.4 (3.1 -- 3.7) [27] |
| HB-EGF | Proheparin-binding EGF-like growth factor | Q99075 | 5.8 (5.6 -- 6.1) [197] | 5.9 (5.6 -- 6.2) [27] |
| GDF-2 | Growth/differentiation factor 2 | Q9UK05 | 4.6 (4.2 -- 4.9) [197] | 4.6 (4.2 -- 4.9) [27] |
| FABP2 | Fatty acid-binding protein, intestinal | P12104 | 9.2 (8.6 -- 9.7) [197] | 9.3 (8.7 -- 9.9) [27] |
| THPO | Thrombopoietin | P40225 | 2.1 (1.9 -- 2.3) [197] | 2.1 (1.9 -- 2.4) [27] |
| MARCO | Macrophage receptor MARCO | Q9UEW3 | 6.2 (6.1 -- 6.4) [197] | 6.2 (6.0 -- 6.4) [27] |
| GT | Gastrotropin | P51161 | 1.9 (1.6 -- 2.4) [197] | 2.0 (1.7 -- 2.4) [27] |
| BNP | Natriuretic peptides B | P16860 | 4.0 (3.0 -- 4.9) [197] | 4.9 (3.9 -- 5.9) [27] |
| MMP-12 | Matrix metalloproteinase-12 | P39900 | 7.8 (7.3 -- 8.3) [197] | 8.0 (7.6 -- 8.6) [27] |
| ACE2 | Angiotensin-converting enzyme 2 | Q9BYF1 | 3.9 (3.5 -- 4.4) [197] | 4.1 (3.7 -- 4.6) [27] |
| PD-L2 | Programmed cell death 1 ligand 2 | Q9BQ51 | 3.1 (2.9 -- 3.3) [197] | 3.2 (3.0 -- 3.5) [27] |
| CTSL1 | Cathepsin L1 | P07711 | 6.1 (5.7 -- 6.4) [197] | 6.2 (5.9 -- 6.6) [27] |
| hOSCAR | Osteoclast-associated immunoglobulin-like receptor | Q8IYS5 | 9.8 (9.6 -- 9.9) [197] | 9.8 (9.7 -- 10.0) [27] |
| TNFRSF13B | Tumor necrosis factor receptor superfamily member 13B | O14836 | 8.4 (8.1 -- 8.7) [197] | 8.6 (8.3 -- 8.9) [27] |
| TGM2 | Protein-glutamine gamma-glutamyltransferase 2 | P21980 | 8.3 (7.8 -- 8.8) [197] | 8.4 (7.9 -- 8.9) [27] |
| LEP | Leptin | P41159 | 6.7 (6.0 -- 7.4) [197] | 6.5 (5.5 -- 7.3) [27] |
| CA5A | Carbonic anhydrase 5A, mitochondrial | P35218 | 2.6 (2.1 -- 3.3) [197] | 2.6 (2.1 -- 3.2) [27] |
| HSP 27 | Heat shock 27 kDa protein | P04792 | 10.2 (9.6 -- 10.6) [197] | 10.3 (9.8 -- 10.6) [27] |
| CD4 | T-cell surface glycoprotein CD4 | P01730 | 4.7 (4.5 -- 4.9) [197] | 4.9 (4.6 -- 5.1) [27] |
| NEMO | NF-kappa-B essential modulator | Q9Y6K9 | 4.2 (3.7 -- 5.0) [197] | 4.4 (3.9 -- 5.3) [27] |
| VEGF-D | Vascular endothelial growth factor D | O43915 | 7.4 (7.2 -- 7.7) [197] | 7.6 (7.3 -- 7.9) [27] |
| PARP-1 | Poly [ADP-ribose] polymerase 1 | P09874 | 3.4 (3.0 -- 3.8) [197] | 3.4 (3.0 -- 3.9) [27] |
| HAOX1 | Hydroxyacid oxidase 1 | Q9UJM8 | 4.6 (3.7 -- 5.7) [197] | 4.6 (3.6 -- 5.7) [27] |
| TNFRSF14 | Tumor necrosis factor receptor superfamily member 14 | Q92956 | 5.0 (4.8 -- 5.3) [22] | 5.2 (4.9 -- 5.6) [3] |
| LDL receptor | Low-density lipoprotein receptor | P01130 | 4.6 (4.2 -- 5.1) [22] | 4.5 (4.0 -- 4.9) [3] |
| ITGB2 | Integrin beta-2 | P05107 | 5.7 (5.5 -- 6.0) [22] | 5.7 (5.4 -- 6.0) [3] |
| IL-17RA | Interleukin-17 receptor A | Q96F46 | 4.2 (3.8 -- 4.5) [22] | 4.2 (3.9 -- 4.5) [3] |
| TNF-R2 | Tumor necrosis factor receptor 2 | P20333 | 4.9 (4.6 -- 5.2) [22] | 5.1 (4.8 -- 5.5) [3] |
| MMP-9 | Matrix metalloproteinase-9 | P14780 | 4.2 (3.7 -- 4.7) [22] | 4.3 (3.8 -- 4.8) [3] |
| EPHB4 | Ephrin type-B receptor 4 | P54760 | 2.3 (2.1 -- 2.5) [22] | 2.4 (2.1 -- 2.6) [3] |
| IL2-RA | Interleukin-2 receptor subunit alpha | P01589 | 4.2 (3.9 -- 4.6) [22] | 4.4 (4.1 -- 4.8) [3] |
| OPG | Osteoprotegerin | O00300 | 3.6 (3.4 -- 3.9) [22] | 3.8 (3.5 -- 4.1) [3] |
| ALCAM | CD166 antigen | Q13740 | 4.9 (4.7 -- 5.1) [22] | 4.9 (4.8 -- 5.1) [3] |
| TFF3 | Trefoil factor 3 | Q07654 | 5.6 (5.3 -- 5.9) [22] | 5.9 (5.5 -- 6.3) [3] |
| SELP | P-selectin | P16109 | 9.1 (8.7 -- 9.5) [22] | 9.1 (8.8 -- 9.6) [3] |
| CSTB | Cystatin-B | P04080 | 5.1 (4.8 -- 5.5) [91] | 5.4 (4.9 -- 5.9) [11] |
| MCP-1 | Monocyte chemotactic protein 1 | P13500 | 3.4 (3.2 -- 3.6) [22] | 3.5 (3.2 -- 3.7) [3] |
| CD163 | Scavenger receptor cysteine-rich type 1 protein M130 | Q86VB7 | 7.9 (7.6 -- 8.2) [22] | 8.0 (7.7 -- 8.3) [3] |
| Gal-3 | Galectin-3 | P17931 | 5.7 (5.4 -- 5.9) [22] | 5.8 (5.5 -- 6.0) [3] |
| GRN | Granulins | P28799 | 3.5 (3.3 -- 3.7) [22] | 3.6 (3.4 -- 3.8) [3] |
| MEPE | Matrix extracellular phosphoglycoprotein | Q9NQ76 | 3.4 (3.1 -- 3.7) [22] | 3.4 (3.1 -- 3.8) [3] |
| BLM hydrolase | Bleomycin hydrolase | Q13867 | 5.7 (5.5 -- 6.0) [161] | 5.8 (5.5 -- 6.0) [13] |
| PLC | Perlecan | P98160 | 6.9 (6.7 -- 7.2) [22] | 7.1 (6.8 -- 7.4) [3] |
| LTBR | Lymphotoxin-beta receptor | P36941 | 3.9 (3.7 -- 4.1) [22] | 4.0 (3.8 -- 4.4) [3] |
| NOTCH-3 | Neurogenic locus notch homolog protein 3 | Q9UM47 | 4.4 (4.1 -- 4.7) [22] | 4.5 (4.3 -- 4.8) [3] |
| TIMP4 | Metalloproteinase inhibitor 4 | Q99727 | 5.2 (4.8 -- 5.5) [22] | 5.3 (5.0 -- 5.7) [3] |
| CNTN1 | Contactin-1 | Q12860 | 3.2 (3.0 -- 3.4) [22] | 3.2 (3.0 -- 3.4) [3] |
| CDH5 | Cadherin-5 | Q9NPY3 | 3.8 (3.5 -- 4.0) [22] | 3.9 (3.6 -- 4.1) [3] |
| TLT-2 | Trem-like transcript 2 protein | Q5T2D2 | 4.2 (3.9 -- 4.5) [22] | 4.2 (3.9 -- 4.6) [3] |
| FABP4 | Fatty acid-binding protein, adipocyte | P15090 | 5.1 (4.5 -- 5.7) [22] | 5.2 (4.6 -- 5.9) [3] |
| TFPI | Tissue factor pathway inhibitor | P10646 | 8.6 (8.3 -- 8.8) [22] | 8.6 (8.5 -- 8.9) [3] |
| PAI | Plasminogen activator inhibitor 1 | P05121 | 6.3 (5.6 -- 6.9) [22] | 6.1 (5.5 -- 6.8) [3] |
| CCL24 | C-C motif chemokine 24 | O00175 | 5.8 (5.2 -- 6.4) [22] | 5.9 (5.2 -- 6.5) [3] |
| TR | Transferrin receptor protein 1 | P02786 | 5.2 (4.8 -- 5.6) [22] | 5.5 (5.0 -- 5.9) [3] |
| TNFRSF10C | Tumor necrosis factor receptor superfamily member 10C | O14798 | 6.1 (5.8 -- 6.4) [22] | 6.1 (5.8 -- 6.4) [3] |
| GDF-15 | Growth/differentiation factor 15 | Q99988 | 5.3 (4.9 -- 5.8) [22] | 5.7 (5.3 -- 6.2) [3] |
| SELE | E-selectin | P16581 | 2.5 (2.1 -- 2.8) [22] | 2.5 (2.1 -- 2.9) [3] |
| AZU1 | Azurocidin | P20160 | 3.0 (2.5 -- 3.7) [22] | 3.0 (2.6 -- 3.7) [3] |
| DLK-1 | Protein delta homolog 1 | P80370 | 5.4 (5.0 -- 5.8) [22] | 5.4 (4.9 -- 5.8) [3] |
| SPON1 | Spondin-1 | Q9HCB6 | 2.3 (2.1 -- 2.5) [22] | 2.4 (2.2 -- 2.7) [3] |
| MPO | Myeloperoxidase | P05164 | 4.4 (4.1 -- 4.7) [22] | 4.4 (4.2 -- 4.8) [3] |
| CXCL16 | C-X-C motif chemokine 16 | Q9H2A7 | 6.3 (6.1 -- 6.5) [22] | 6.3 (6.1 -- 6.6) [3] |
| IL-6RA | Interleukin-6 receptor subunit alpha | P08887 | 11.0 (10.7 -- 11.3) [22] | 11.0 (10.7 -- 11.3) [3] |
| RETN | Resistin | Q9HD89 | 6.9 (6.5 -- 7.2) [22] | 7.0 (6.6 -- 7.4) [3] |
| IGFBP-1 | Insulin-like growth factor-binding protein 1 | P08833 | 5.3 (4.3 -- 6.2) [22] | 5.9 (5.0 -- 6.7) [3] |
| CHIT1 | Chitotriosidase-1 | Q13231 | 3.4 (2.6 -- 4.1) [22] | 3.5 (2.6 -- 4.3) [3] |
| TR-AP | Tartrate-resistant acid phosphatase type 5 | P13686 | 5.0 (4.7 -- 5.3) [22] | 5.0 (4.6 -- 5.2) [3] |
| CCL22 | C-C motif chemokine 22 | O00175 | 2.3 (2.0 -- 2.8) [22] | 2.3 (1.9 -- 2.7) [3] |
| PSP-D | Pulmonary surfactant-associated protein D | P35247 | 2.8 (2.3 -- 3.3) [22] | 2.8 (2.3 -- 3.4) [3] |
| PI3 | Elafin | P19957 | 4.2 (3.7 -- 4.6) [86] | 4.4 (4.0 -- 5.0) [13] |
| Ep-CAM | Epithelial cell adhesion molecule | P16422 | 4.0 (3.5 -- 4.7) [22] | 4.0 (3.5 -- 4.5) [3] |
| AP-N | Aminopeptidase N | P15144 | 5.1 (4.9 -- 5.3) [22] | 5.2 (5.0 -- 5.4) [3] |
| AXL | Tyrosine-protein kinase receptor UFO | P30530 | 8.1 (7.9 -- 8.4) [22] | 8.2 (8.0 -- 8.5) [3] |
| IL-1RT1 | Interleukin-1 receptor type 1 | P14778 | 6.6 (6.4 -- 6.8) [22] | 6.7 (6.5 -- 6.9) [3] |
| MMP-2 | Matrix metalloproteinase-2 | P08253 | 4.1 (3.9 -- 4.4) [22] | 4.3 (3.9 -- 4.5) [3] |
| FAS | Tumor necrosis factor receptor superfamily member 6 | P25445 | 5.0 (4.8 -- 5.2) [22] | 5.1 (4.9 -- 5.3) [3] |
| MB | Myoglobin | P02144 | 7.3 (6.9 -- 7.7) [22] | 7.4 (7.0 -- 7.7) [3] |
| TNFSF13B | Tumor necrosis factor ligand superfamily member 13B | Q9Y275 | 6.5 (6.3 -- 6.8) [22] | 6.6 (6.3 -- 7.0) [3] |
| PRTN3 | Myeloblastin | P24158 | 4.7 (4.4 -- 5.1) [22] | 4.9 (4.5 -- 5.3) [3] |
| PCSK9 | Proprotein convertase subtilisin/kexin type 9 | Q8NBP7 | 2.9 (2.6 -- 3.2) [22] | 2.9 (2.6 -- 3.2) [3] |
| U-PAR | Urokinase plasminogen activator surface receptor | Q03405 | 4.8 (4.5 -- 5.1) [22] | 5.0 (4.7 -- 5.3) [3] |
| OPN | Osteopontin | P10451 | 5.4 (5.0 -- 5.7) [22] | 5.6 (5.2 -- 6.0) [3] |
| CTSD | Cathepsin D | P07339 | 4.3 (4.0 -- 4.7) [22] | 4.4 (4.1 -- 4.8) [3] |
| PGLYRP1 | Peptidoglycan recognition protein 1 | O75594 | 7.7 (7.4 -- 8.0) [22] | 7.9 (7.5 -- 8.2) [3] |
| CPA1 | Carboxypeptidase A1 | P15085 | 4.6 (4.2 -- 5.1) [22] | 4.6 (4.2 -- 5.1) [3] |
| JAM-A | Junctional adhesion molecule A | Q9Y624 | 4.4 (4.1 -- 4.7) [22] | 4.6 (4.3 -- 5.0) [3] |
| Gal-4 | Galectin-4 | P56470 | 3.8 (3.4 -- 4.1) [22] | 3.9 (3.5 -- 4.3) [3] |
| IL-1RT2 | Interleukin-1 receptor type 2 | P27930 | 5.2 (5.0 -- 5.5) [22] | 5.2 (5.0 -- 5.5) [3] |
| SHPS-1 | Tyrosine-protein phosphatase non- receptor type substrate 1 | P78324 | 4.0 (3.7 -- 4.3) [22] | 4.1 (3.8 -- 4.4) [3] |
| CCL15 | C-C motif chemokine 15 | Q16663 | 7.3 (7.0 -- 7.7) [22] | 7.5 (7.2 -- 8.0) [3] |
| CASP-3 | Caspase-3 | P42574 | 6.5 (5.7 -- 7.8) [22] | 6.7 (5.9 -- 7.9) [3] |
| uPA | Urokinase-type plasminogen activator | P00749 | 5.0 (4.8 -- 5.2) [22] | 5.1 (4.8 -- 5.3) [3] |
| CPB1 | Carboxypeptidase B | P15086 | 4.1 (3.7 -- 4.6) [22] | 4.2 (3.7 -- 4.7) [3] |
| CHI3L1 | Chitinase-3-like protein 1 | P36222 | 7.4 (6.8 -- 8.2) [22] | 7.8 (7.1 -- 8.5) [3] |
| ST2 | ST2 protein | Q01638 | 4.2 (3.9 -- 4.5) [22] | 4.4 (4.0 -- 4.8) [3] |
| t-PA | Tissue-type plasminogen activator | P00750 | 6.3 (5.9 -- 6.7) [22] | 6.3 (5.9 -- 6.7) [3] |
| SCGB3A2 | Secretoglobin family 3A member 2 | Q96PL1 | 2.6 (2.2 -- 3.2) [22] | 2.9 (2.3 -- 3.5) [3] |
| EGFR | Epidermal growth factor receptor | P00533 | 1.9 (1.8 -- 2.1) [22] | 1.8 (1.7 -- 2.0) [3] |
| IGFBP-7 | Insulin-like growth factor-binding protein 7 | Q16270 | 4.6 (4.3 -- 4.9) [22] | 4.8 (4.5 -- 5.2) [3] |
| CD93 | Complement component C1q receptor | Q9NPY3 | 9.9 (9.6 -- 10.1) [22] | 10.0 (9.7 -- 10.2) [3] |
| IL-18BP | Interleukin-18-binding protein | O95998 | 6.7 (6.5 -- 7.0) [22] | 6.9 (6.6 -- 7.2) [3] |
| COL1A1 | Collagen alpha-1(I) chain | P02452 | 2.5 (2.2 -- 2.8) [22] | 2.6 (2.3 -- 2.8) [3] |
| PON3 | Paraoxonase | Q15166 | 5.7 (5.3 -- 6.1) [22] | 5.6 (5.2 -- 6.0) [3] |
| CTSZ | Cathepsin Z | Q9UBR2 | 5.0 (4.8 -- 5.3) [22] | 5.1 (4.8 -- 5.4) [3] |
| MMP-3 | Matrix metalloproteinase-3 | P08254 | 7.5 (7.0 -- 7.9) [22] | 7.6 (7.1 -- 8.1) [3] |
| RARRES2 | Retinoic acid receptor responder protein 2 | Q99969 | 12.1 (11.9 -- 12.2) [22] | 12.1 (11.9 -- 12.3) [3] |
| ICAM-2 | Intercellular adhesion molecule 2 | P13598 | 5.3 (5.0 -- 5.5) [22] | 5.4 (5.1 -- 5.6) [3] |
| KLK6 | Kallikrein-6 | Q92876 | 3.5 (3.5 -- 3.6) [22] | 3.5 (3.5 -- 3.7) [3] |
| PDGF subunit A | Platelet-derived growth factor subunit A | P04085 | 3.1 (2.5 -- 3.7) [22] | 3.0 (2.4 -- 3.7) [3] |
| TNF-R1 | Tumor necrosis factor receptor 1 | P19438 | 5.3 (5.0 -- 5.6) [22] | 5.5 (5.2 -- 5.9) [3] |
| IGFBP-2 | Insulin-like Growth Factor-Binding Protein 2 | P18065 | 8.0 (7.5 -- 8.5) [22] | 8.4 (7.9 -- 8.9) [3] |
| vWF | von Willebrand factor | P04275 | 6.4 (5.8 -- 7.0) [22] | 6.6 (5.9 -- 7.2) [3] |
| PECAM-1 | Platelet endothelial cell adhesion molecule | P16284 | 4.8 (4.5 -- 5.0) [22] | 4.8 (4.6 -- 5.1) [3] |
| NT-proBNP | N-terminal prohormone brain natriuretic peptide | NA | 2.8 (2.1 -- 3.4) [22] | 3.5 (2.8 -- 4.3) [3] |
| CCL16 | C-C motif chemokine 16 | O15467 | 6.1 (5.8 -- 6.5) [22] | 6.2 (5.8 -- 6.6) [3] |
| IL-8 | Interleukin-8 | P10145 | 6.3 (5.9 -- 6.7) [189] | 6.5 (6.2 -- 7.0) [21] |
| VEGF-A | Vascular endothelial growth factor A | P15692 | 10.1 (9.9 -- 10.4) [189] | 10.2 (10.0 -- 10.6) [21] |
| BDNF | Brain-derived neurotrophic factor | P23560 | 2.4 (2.4 -- 6.2) [189] | 2.4 (2.4 -- 5.0) [21] |
| MCP-3 | Monocyte chemotactic protein 3 | P80098 | 2.0 (1.9 -- 2.4) [189] | 2.2 (1.9 -- 2.6) [21] |
| hGDNF | Glial cell line-derived neurotrophic factor | P39905 | 2.2 (2.0 -- 2.5) [189] | 2.4 (2.1 -- 2.7) [21] |
| CDCP1 | CUB domain-containing protein 1 | Q9H5V8 | 3.0 (2.6 -- 3.4) [189] | 3.2 (2.8 -- 3.7) [21] |
| CD244 | Natural killer cell receptor 2B4 | Q9BZW8 | 6.0 (5.8 -- 6.2) [189] | 6.1 (5.8 -- 6.3) [21] |
| IL-7 | Interleukin-7 | P13232 | 3.3 (2.8 -- 3.8) [255] | 3.2 (2.7 -- 3.8) [27] |
| LAP TGF-beta-1 | Latency-associated peptide transforming growth factor beta 1 | P01137 | 6.3 (6.1 -- 6.6) [189] | 6.4 (6.1 -- 6.8) [21] |
| IL-17C | Interleukin-17C | Q9P0M4 | 1.5 (1.5 -- 1.8) [189] | 1.6 (1.5 -- 1.9) [21] |
| IL-17A | Interleukin-17A | Q16552 | 0.5 (0.4 -- 0.8) [189] | 0.6 (0.4 -- 0.9) [21] |
| CXCL11 | C-X-C motif chemokine 11 | O14625 | 6.9 (6.4 -- 7.5) [189] | 7.1 (6.6 -- 7.8) [21] |
| AXIN1 | Axin-1 | O15169 | 1.6 (1.4 -- 2.5) [189] | 1.7 (1.4 -- 2.7) [21] |
| TRAIL | TNF-related apoptosis-inducing ligand | P50591 | 7.6 (7.4 -- 7.8) [189] | 7.6 (7.4 -- 7.8) [21] |
| IL-20RA | Interleukin-20 receptor subunit alpha | Q9UHF4 | 0.9 (0.9 -- 0.9) [189] | 0.9 (0.9 -- 0.9) [21] |
| CXCL9 | C-X-C motif chemokine 9 | Q07325 | 8.5 (8.0 -- 9.1) [189] | 8.8 (8.2 -- 9.5) [21] |
| CST5 | Cystatin D | P28325 | 7.0 (6.6 -- 7.4) [189] | 7.2 (6.8 -- 7.6) [21] |
| IL-2RB | Interleukin-2 receptor subunit beta | P14784 | 0.8 (0.8 -- 0.8) [189] | 0.8 (0.8 -- 0.8) [21] |
| IL-1 alpha | Interleukin-1 alpha | P01583 | 1.7 (1.7 -- 1.7) [189] | 1.7 (1.7 -- 1.7) [21] |
| OSM | Oncostatin-M | P13725 | 2.6 (2.1 -- 3.1) [189] | 2.8 (2.2 -- 3.3) [21] |
| IL-2 | Interleukin-2 | P60568 | 1.4 (1.4 -- 1.4) [189] | 1.4 (1.4 -- 1.4) [21] |
| TSLP | Thymic stromal lymphopoietin | Q969D9 | 1.1 (1.1 -- 1.1) [189] | 1.1 (1.1 -- 1.1) [21] |
| CCL4 | C-C motif chemokine 4 | P13236 | 5.8 (5.5 -- 6.2) [189] | 5.9 (5.6 -- 6.2) [21] |
| CD6 | T cell surface glycoprotein CD6 isoform | Q8WWJ7 | 4.0 (3.7 -- 4.2) [189] | 4.0 (3.7 -- 4.3) [21] |
| SLAMF1 | Signaling lymphocytic activation molecule | Q13291 | 3.5 (3.2 -- 3.8) [189] | 3.7 (3.3 -- 4.0) [21] |
| TGF-alpha | Transforming growth factor alpha | P01135 | 1.2 (0.9 -- 1.4) [189] | 1.3 (1.1 -- 1.6) [21] |
| MCP-4 | Monocyte chemotactic protein 4 | Q99616 | 2.3 (1.9 -- 2.7) [189] | 2.3 (2.0 -- 2.7) [21] |
| CCL11 | Eotaxin-1 | P51671 | 8.1 (7.9 -- 8.4) [189] | 8.2 (7.9 -- 8.5) [21] |
| TNFSF14 | Tumor necrosis factor ligand superfamily member 14 | O43557 | 2.6 (2.3 -- 3.0) [189] | 2.7 (2.3 -- 3.1) [21] |
| IL-10RA | Interleukin-10 receptor subunit alpha | Q13651 | 0.9 (0.9 -- 0.9) [189] | 0.9 (0.9 -- 1.0) [21] |
| FGF-5 | Fibroblast growth factor 5 | Q8NF90 | 1.9 (1.7 -- 2.1) [189] | 1.9 (1.8 -- 2.1) [21] |
| MMP-1 | Matrix metalloproteinase-1 | P03956 | 7.5 (6.8 -- 8.3) [189] | 7.7 (6.8 -- 8.4) [21] |
| LIF-R | Leukemia inhibitory factor receptor | P42702 | 3.9 (3.8 -- 4.1) [189] | 4.1 (3.9 -- 4.3) [21] |
| CCL19 | C-C motif chemokine 19 | Q99731 | 9.4 (9.0 -- 10.0) [189] | 9.6 (9.0 -- 10.2) [21] |
| IL-15RA | Interleukin-15 receptor subunit alpha | Q13261 | 1.1 (0.9 -- 1.2) [189] | 1.2 (1.0 -- 1.4) [21] |
| IL-10RB | Interleukin-10 receptor subunit beta | Q08334 | 6.7 (6.5 -- 6.9) [189] | 6.8 (6.6 -- 7.1) [21] |
| IL-22 RA1 | Interleukin-22 receptor subunit alpha-1 | Q8N6P7 | 2.3 (2.3 -- 2.3) [189] | 2.3 (2.3 -- 2.3) [21] |
| IL-18R1 | Interleukin-18 receptor 1 | Q13478 | 7.5 (7.2 -- 7.8) [189] | 7.6 (7.3 -- 7.9) [21] |
| PD-L1 | Programmed cell death 1 ligand 1 | Q9NZQ7 | 5.0 (4.7 -- 5.3) [189] | 5.1 (4.9 -- 5.4) [21] |
| Beta-NGF | Beta-nerve growth factor | P01138 | 1.6 (1.4 -- 1.8) [189] | 1.7 (1.5 -- 1.9) [21] |
| CXCL5 | C-X-C motif chemokine 5 | P42830 | 10.7 (9.5 -- 11.7) [189] | 10.4 (9.3 -- 11.6) [21] |
| TRANCE | TNF-related activation-induced cytokine | O14788 | 4.6 (4.2 -- 5.0) [189] | 4.5 (4.0 -- 5.1) [21] |
| HGF | Hepatocyte growth factor | P14210 | 7.5 (7.2 -- 7.8) [189] | 7.7 (7.4 -- 8.0) [21] |
| IL-12B | Interleukin-12 subunit beta | P29460 | 4.8 (4.4 -- 5.3) [189] | 5.0 (4.5 -- 5.4) [21] |
| IL-24 | Interleukin-24 | Q13007 | 0.4 (0.4 -- 0.4) [189] | 0.4 (0.4 -- 0.4) [21] |
| IL-13 | Interleukin-13 | P35225 | 1.1 (1.1 -- 1.1) [189] | 1.1 (1.1 -- 1.1) [21] |
| ARTN | Artemin | Q5T4W7 | 0.2 (0.2 -- 0.2) [189] | 0.2 (0.2 -- 0.2) [21] |
| MMP-10 | Matrix metalloproteinase-10 | P09238 | 9.2 (8.9 -- 9.7) [189] | 9.4 (8.9 -- 9.8) [21] |
| IL-10 | Interleukin-10 | P22301 | 4.3 (4.0 -- 4.6) [189] | 4.3 (4.0 -- 4.7) [21] |
| TNF | Tumor necrosis factor | P01375 | 0.9 (0.9 -- 0.9) [189] | 0.9 (0.9 -- 0.9) [21] |
| CCL23 | C-C motif chemokine 23 | P55773 | 10.0 (9.7 -- 10.4) [189] | 10.2 (9.8 -- 10.5) [21] |
| CD5 | T-cell surface glycoprotein CD5 | P06127 | 3.4 (3.2 -- 3.7) [189] | 3.5 (3.2 -- 3.8) [21] |
| Flt3L | Fms-related tyrosine kinase 3 ligand | P49771 | 9.3 (9.1 -- 9.6) [189] | 9.3 (9.0 -- 9.6) [21] |
| CXCL6 | C-X-C motif chemokine 6 | P80162 | 7.3 (6.8 -- 7.9) [189] | 7.4 (6.8 -- 7.9) [21] |
| CXCL10 | C-X-C motif chemokine 10 | P02778 | 10.2 (9.8 -- 10.8) [189] | 10.5 (10.0 -- 11.1) [21] |
| 4E-BP1 | Eukaryotic translation initiation factor 4E-binding protein 1 | Q13541 | 8.4 (7.8 -- 9.1) [189] | 8.7 (8.1 -- 9.4) [21] |
| IL-20 | Interleukin-20 | Q9NYY1 | 0.8 (0.8 -- 0.8) [189] | 0.8 (0.8 -- 0.8) [21] |
| SIRT2 | SIR2-like protein 2 | Q8IXJ6 | 4.1 (3.4 -- 5.3) [189] | 4.3 (3.6 -- 5.6) [21] |
| CCL28 | C-C motif chemokine 28 | Q9NRJ3 | 1.0 (0.8 -- 1.2) [189] | 1.1 (0.9 -- 1.3) [21] |
| DNER | Delta and Notch-like epidermal growth factor-related receptor | Q8NFT8 | 7.1 (6.9 -- 7.3) [189] | 7.1 (6.9 -- 7.3) [21] |
| EN-RAGE | Protein S100-A12 | P80511 | 2.7 (2.1 -- 3.2) [189] | 2.9 (2.3 -- 3.6) [21] |
| CD40 | CD40L receptor | P25942 | 9.3 (9.1 -- 9.6) [189] | 9.5 (9.2 -- 9.9) [21] |
| IL-33 | Interleukin-33 | O95760 | 1.7 (1.7 -- 1.7) [189] | 1.7 (1.7 -- 1.7) [21] |
| IFN-gamma | Interferon gamma | P01579 | 1.1 (1.1 -- 1.1) [189] | 1.1 (1.1 -- 1.1) [21] |
| FGF-19 | Fibroblast growth factor 19 | O95750 | 8.1 (7.4 -- 8.8) [189] | 8.1 (7.3 -- 8.9) [21] |
| IL-4 | Interleukin-4 | P05112 | 1.5 (1.5 -- 1.5) [189] | 1.5 (1.5 -- 1.5) [21] |
| LIF | Leukemia inhibitory factor | P15018 | 0.6 (0.6 -- 0.6) [189] | 0.6 (0.6 -- 0.6) [21] |
| NRTN | Neurturin | Q99748 | 1.2 (1.2 -- 1.2) [189] | 1.2 (1.2 -- 1.2) [21] |
| MCP-2 | Monocyte chemotactic protein 2 | P80075 | 9.0 (8.6 -- 9.4) [189] | 9.1 (8.6 -- 9.5) [21] |
| CASP-8 | Caspase 8 | Q14790 | 2.8 (2.4 -- 3.5) [189] | 2.9 (2.4 -- 3.7) [21] |
| CCL25 | C-C motif chemokine 25 | O15444 | 6.9 (6.5 -- 7.3) [189] | 7.1 (6.7 -- 7.5) [21] |
| CX3CL1 | Fractalkine | P78423 | 5.7 (5.5 -- 6.0) [189] | 5.9 (5.6 -- 6.2) [21] |
| TNFRSF9 | Tumor necrosis factor receptor superfamily member 9 | Q07011 | 6.5 (6.1 -- 6.8) [189] | 6.7 (6.3 -- 7.1) [21] |
| NT-3 | Neurotrophin-3 | P20783 | 1.9 (1.7 -- 2.2) [189] | 2.0 (1.7 -- 2.4) [21] |
| TWEAK | Tumor necrosis factor (Ligand) superfamily, member 12 | Q4ACW9 | 8.3 (8.1 -- 8.5) [189] | 8.2 (8.0 -- 8.4) [21] |
| CCL20 | C-C motif chemokine 20 | P78556 | 6.6 (6.0 -- 7.3) [189] | 6.8 (6.2 -- 7.6) [21] |
| ST1A1 | Sulfotransferase 1A1 | P50225 | 1.3 (0.5 -- 2.6) [189] | 1.5 (0.6 -- 2.8) [21] |
| STAMPB | STAM-binding protein | O95630 | 3.3 (2.9 -- 4.1) [189] | 3.5 (3.0 -- 4.2) [21] |
| IL-5 | Interleukin-5 | P05113 | 1.6 (1.6 -- 1.6) [189] | 1.6 (1.6 -- 1.6) [21] |
| ADA | Adenosine Deaminase | P00813 | 4.1 (3.9 -- 4.4) [189] | 4.2 (3.9 -- 4.5) [21] |
| TNFB | TNF-beta | P01374 | 3.1 (2.8 -- 3.3) [189] | 3.1 (2.8 -- 3.3) [21] |
| CSF-1 | Macrophage colony-stimulating factor 1 | P09603 | 8.0 (7.8 -- 8.1) [189] | 8.1 (7.9 -- 8.2) [21] |

Continuous variables presented as median (Q1-Q3). Number of missing values presented in [n].

The CVDII panel was used for biomarkers ranging from BMP-6 to HAOX1; CVDIII panel for TNFRSF14 to CCL16; and Inflammation panel from IL-8 to CSF-1.

# **Supplemental Table 1B**

Baseline levels of biomarkers expressed in NPX values (arbitrary units) for cases and controls in the validation cohort

| Variable |  | UniProt No. | No | CV death |
| --- | --- | --- | --- | --- |
| BMP-6 | Bone morphogenetic protein 6 | P22004 | 4.8 (4.5 -- 5.1) | 4.9 (4.6 -- 5.3) |
| ANG-1 | Angiopoietin-1 | Q15389 | 8.0 (7.1 -- 8.9) | 7.8 (6.9 -- 8.6) |
| ADM | ADM | P35318 | 7.3 (6.9 -- 7.6) | 7.5 (7.2 -- 7.8) |
| CD40-L | CD40 ligand | P29965 | 3.6 (3.0 -- 4.6) | 3.7 (3.1 -- 4.7) |
| SLAMF7 | SLAM family member 7 | Q9NQ25 | 4.0 (3.6 -- 4.4) [1] | 4.2 (3.8 -- 4.6) [0] |
| PlGF | Placenta growth factor | P49763 | 7.9 (7.7 -- 8.2) | 8.1 (7.9 -- 8.4) |
| ADAM-TS13 | A disintegrin and metalloproteinase with thrombospondin motifs 13 | Q76LX8 | 5.9 (5.8 -- 6.0) | 5.9 (5.7 -- 6.0) |
| Protein BOC | Brother of CDO | Q9BWV1 | 3.9 (3.7 -- 4.1) | 3.9 (3.8 -- 4.2) |
| IL-4RA | Interleukin-4 receptor subunit alpha | P24394 | 1.9 (1.6 -- 2.1) | 2.0 (1.7 -- 2.3) |
| SRC | Proto-oncogene tyrosine-protein kinase Src | P12931 | 5.1 (4.0 -- 6.8) | 5.3 (4.0 -- 6.9) |
| IL-1ra | Interleukin-1 receptor antagonist protein | P18510 | 4.6 (4.1 -- 5.1) | 4.8 (4.3 -- 5.4) |
| IL-6 | Interleukin-6 | P05231 | 3.5 (3.0 -- 4.1) | 4.1 (3.4 -- 4.8) |
| TNFRSF10A | Tumor necrosis factor receptor superfamily member 10A | O00220 | 3.8 (3.5 -- 4.0) | 4.0 (3.7 -- 4.3) |
| STK4 | Serine/threonine-protein kinase 4 | Q13043 | 2.4 (1.3 -- 4.1) | 2.5 (1.3 -- 4.2) |
| IDUA | Alpha-L-iduronidase | P35475 | 5.4 (5.0 -- 5.7) | 5.4 (5.1 -- 5.7) |
| TNFRSF11A | Tumor necrosis factor receptor superfamily member 11A | Q9Y6Q6 | 5.8 (5.5 -- 6.1) | 6.0 (5.7 -- 6.5) |
| PAR-1 | Proteinase-activated receptor 1 | P25116 | 8.6 (8.3 -- 8.9) | 8.8 (8.4 -- 9.1) |
| TRAIL-R2 | TNF-related apoptosis-inducing ligand receptor 2 | O14763 | 5.9 (5.7 -- 6.2) | 6.2 (6.0 -- 6.6) |
| PRSS27 | Serine protease 27 | Q9BQR3 | 8.3 (8.0 -- 8.6) | 8.3 (8.0 -- 8.7) |
| TIE2 | Angiopoietin-1 receptor | Q02763 | 7.2 (7.0 -- 7.3) | 7.2 (7.0 -- 7.4) |
| TF | Tissue factor | P13726 | 5.2 (5.0 -- 5.4) | 5.3 (5.1 -- 5.5) |
| IL1RL2 | Interleukin-1 receptor-like 2 | Q9HB29 | 4.5 (4.2 -- 4.8) | 4.4 (4.1 -- 4.7) |
| PDGF subunit B | Platelet-derived growth factor subunit B | P01127 | 8.7 (7.8 -- 9.6) | 8.7 (7.8 -- 9.5) |
| IL-27 | Interleukin-27 | Q8NEV9, Q14213 | 5.8 (5.5 -- 6.1) | 5.9 (5.5 -- 6.2) |
| IL-17D | Interleukin-17D | Q8TAD2 | 2.7 (2.4 -- 2.8) | 2.7 (2.4 -- 2.9) |
| CXCL1 | C-X-C motif chemokine 1 | P09341 | 8.8 (7.9 -- 9.7) | 8.9 (7.9 -- 9.6) |
| LOX-1 | Lectin-like oxidized LDL receptor 1 | P78380 | 6.6 (6.2 -- 7.0) | 6.7 (6.3 -- 7.1) |
| Gal-9 | Galectin-9 | O00182 | 7.8 (7.5 -- 8.0) | 7.9 (7.7 -- 8.2) |
| GIF | Gastric intrinsic factor | P27352 | 7.8 (7.1 -- 8.4) | 7.6 (6.9 -- 8.2) |
| SCF | Stem cell factor | P21583 | 8.7 (8.5 -- 9.0) | 8.7 (8.3 -- 9.0) |
| IL-18 | Interleukin-18 | Q14116 | 8.5 (8.2 -- 8.9) | 8.6 (8.3 -- 9.0) |
| FGF-21 | Fibroblast growth factor 21 | Q9NSA1 | 7.9 (7.0 -- 8.7) | 8.3 (7.3 -- 9.3) |
| PIgR | Polymeric immunoglobulin receptor | P01833 | 6.3 (6.2 -- 6.4) | 6.4 (6.3 -- 6.4) |
| RAGE | Receptor for advanced glycosylation end products | Q15109 | 13.3 (13.0 -- 13.6) | 13.5 (13.1 -- 13.8) |
| SOD2 | Superoxide dismutase [Mn], mitochondrial | P04179 | 9.4 (9.4 -- 9.5) | 9.4 (9.4 -- 9.5) |
| CTRC | Chymotrypsin C | Q99895 | 10.2 (9.7 -- 10.7) | 10.2 (9.7 -- 10.8) |
| FGF-23 | Fibroblast growth factor 23 | Q9GZV9 | 4.7 (4.3 -- 5.2) | 5.1 (4.6 -- 6.0) |
| SPON2 | Spondin-2 | Q9BUD6 | 8.2 (8.0 -- 8.3) | 8.3 (8.1 -- 8.4) |
| GH | Growth hormone | P01241 | 7.4 (5.9 -- 8.9) | 8.1 (6.7 -- 9.2) |
| FS | Follistatin | P19883 | 11.4 (11.1 -- 11.7) | 11.5 (11.2 -- 11.8) |
| GLO1 | Lactoylglutathione lyase | Q04760 | 4.3 (3.8 -- 4.9) | 4.4 (3.9 -- 4.9) |
| CD84 | SLAM family member 5 | Q9UIB8 | 4.1 (3.8 -- 4.4) | 4.2 (3.9 -- 4.5) |
| PAPPA | Pappalysin-1 | Q13219 | 3.4 (3.0 -- 3.8) | 3.5 (3.0 -- 3.9) |
| SERPINA12 | Serpin A12 | Q8IW75 | 2.5 (2.0 -- 3.2) | 2.7 (2.0 -- 3.3) |
| REN | Renin | P00797 | 6.9 (6.2 -- 7.6) | 7.4 (6.6 -- 8.2) |
| DECR1 | 2,4-dienoyl-CoA reductase, mitochondrial | Q16698 | 3.7 (3.1 -- 4.6) | 3.8 (3.2 -- 4.8) |
| MERTK | Tyrosine-protein kinase Mer | Q12866 | 6.0 (5.7 -- 6.2) | 6.0 (5.8 -- 6.4) |
| TIM | T-cell immunoglobulin mucin receptor 1 | Q96D42 | 7.5 (6.9 -- 7.9) | 7.8 (7.3 -- 8.4) |
| THBS2 | Thrombospondin-2 | P35442 | 5.6 (5.4 -- 5.8) | 5.7 (5.5 -- 5.8) |
| TM | Thrombomodulin | P07204 | 10.3 (10.0 -- 10.5) | 10.4 (10.1 -- 10.6) |
| VSIG2 | V-set and immunoglobulin domain-containing protein 2 | Q96IQ7 | 4.8 (4.5 -- 5.2) | 5.0 (4.6 -- 5.5) |
| AMBP | Protein AMBP | P02760 | 7.5 (7.3 -- 7.6) | 7.5 (7.3 -- 7.7) |
| PRELP | Prolargin | P51888 | 8.1 (8.0 -- 8.3) [1] | 8.2 (8.1 -- 8.4) [0] |
| HO-1 | Heme oxygenase 1 | P09601 | 11.4 (11.1 -- 11.6) | 11.4 (11.1 -- 11.6) |
| XCL1 | Lymphotactin | P47992 | 5.0 (4.6 -- 5.4) | 5.0 (4.6 -- 5.5) |
| IL16 | Pro-interleukin-16 | Q14005 | 6.4 (6.1 -- 6.7) | 6.5 (6.1 -- 6.9) |
| SORT1 | Sortilin | Q99523 | 8.5 (8.3 -- 8.7) | 8.5 (8.4 -- 8.7) |
| CEACAM8 | Carcinoembryonic antigenrelated cell adhesion molecule 8 | P31997 | 4.3 (3.9 -- 4.7) | 4.5 (4.1 -- 4.9) |
| PTX3 | Pentraxin-related protein PTX3 | P26022 | 4.3 (4.0 -- 4.7) | 4.4 (4.1 -- 4.8) |
| PSGL-1 | P-selectin glycoprotein ligand 1 | Q14242 | 3.8 (3.7 -- 4.0) | 3.8 (3.6 -- 4.0) |
| CCL17 | C-C motif chemokine 17 | Q92583 | 7.7 (7.1 -- 8.5) | 7.7 (7.2 -- 8.6) |
| CCL3 | C-C motif chemokine 3 | P10147 | 6.3 (5.9 -- 6.7) | 6.5 (6.2 -- 6.9) |
| MMP-7 | Matrix metalloproteinase-7 | P09237 | 10.2 (9.8 -- 10.5) | 10.3 (9.9 -- 10.6) |
| Ig G Fc receptor II-b | Low affinity immunoglobulin gamma Fc region receptor II-b | P31994 | 3.2 (2.5 -- 3.8) | 3.5 (2.8 -- 4.0) |
| ITGB1BP2 | Melusin | Q9UKP3 | 2.7 (2.5 -- 4.2) | 2.9 (2.5 -- 4.5) |
| DCN | Decorin | P07585 | 4.9 (4.7 -- 5.1) | 5.0 (4.8 -- 5.2) |
| Dkk-1 | Dickkopf-related protein 1 | O94907 | 8.4 (8.1 -- 8.9) | 8.5 (8.2 -- 9.0) |
| LPL | Lipoprotein lipase | P06858 | 9.5 (9.1 -- 9.8) | 9.5 (9.1 -- 9.7) |
| PRSS8 | Prostasin | Q16651 | 8.5 (8.2 -- 8.7) | 8.6 (8.3 -- 8.8) |
| AGRP | Agouti-related protein | O00253 | 5.0 (4.7 -- 5.3) | 5.2 (4.9 -- 5.6) |
| HB-EGF | Proheparin-binding EGF-like growth factor | Q99075 | 5.3 (5.0 -- 5.7) | 5.3 (5.0 -- 5.7) |
| GDF-2 | Growth/differentiation factor 2 | Q9UK05 | 9.0 (8.6 -- 9.3) | 9.0 (8.6 -- 9.3) |
| FABP2 | Fatty acid-binding protein, intestinal | P12104 | 8.8 (8.2 -- 9.4) | 8.9 (8.4 -- 9.6) |
| THPO | Thrombopoietin | P40225 | 2.7 (2.5 -- 3.0) | 2.7 (2.5 -- 3.0) |
| MARCO | Macrophage receptor MARCO | Q9UEW3 | 6.3 (6.1 -- 6.4) | 6.3 (6.1 -- 6.4) |
| GT | Gastrotropin | P51161 | 1.9 (1.5 -- 2.4) | 2.0 (1.5 -- 2.5) |
| BNP | Natriuretic peptides B | P16860 | 5.3 (4.0 -- 6.3) | 6.3 (4.9 -- 7.4) |
| MMP-12 | Matrix metalloproteinase-12 | P39900 | 7.0 (6.5 -- 7.5) | 7.3 (6.9 -- 7.9) |
| ACE2 | Angiotensin-converting enzyme 2 | Q9BYF1 | 4.4 (3.9 -- 4.8) | 4.5 (4.1 -- 5.1) |
| PD-L2 | Programmed cell death 1 ligand 2 | Q9BQ51 | 3.1 (2.9 -- 3.3) | 3.3 (3.0 -- 3.6) |
| CTSL1 | Cathepsin L1 | P07711 | 6.9 (6.7 -- 7.1) | 7.1 (6.8 -- 7.4) |
| hOSCAR | Osteoclast-associated immunoglobulin-like receptor | Q8IYS5 | 10.4 (10.2 -- 10.5) | 10.5 (10.3 -- 10.6) |
| TNFRSF13B | Tumor necrosis factor receptor superfamily member 13B | O14836 | 10.1 (9.9 -- 10.4) | 10.3 (10.0 -- 10.6) |
| TGM2 | Protein-glutamine gamma-glutamyltransferase 2 | P21980 | 8.7 (8.3 -- 9.2) | 8.8 (8.4 -- 9.3) |
| LEP | Leptin | P41159 | 7.0 (6.3 -- 7.7) | 7.0 (6.1 -- 7.9) |
| CA5A | Carbonic anhydrase 5A, mitochondrial | P35218 | 2.2 (1.7 -- 2.8) | 2.3 (1.7 -- 3.1) |
| HSP 27 | Heat shock 27 kDa protein | P04792 | 9.1 (8.5 -- 9.6) | 9.4 (8.8 -- 9.7) |
| CD4 | T-cell surface glycoprotein CD4 | P01730 | 5.3 (5.1 -- 5.5) | 5.5 (5.3 -- 5.7) |
| NEMO | NF-kappa-B essential modulator | Q9Y6K9 | 3.2 (2.7 -- 4.0) | 3.5 (2.8 -- 4.3) |
| VEGF-D | Vascular endothelial growth factor D | O43915 | 7.6 (7.4 -- 7.9) | 7.8 (7.5 -- 8.0) |
| PARP-1 | Poly [ADP-ribose] polymerase 1 | P09874 | 3.0 (2.6 -- 3.3) | 3.1 (2.7 -- 3.5) |
| HAOX1 | Hydroxyacid oxidase 1 | Q9UJM8 | 5.4 (4.4 -- 6.4) | 5.2 (4.3 -- 6.5) |
| TNFRSF14 | Tumor necrosis factor receptor superfamily member 14 | Q92956 | 4.5 (4.3 -- 4.8) | 4.8 (4.5 -- 5.1) |
| LDL receptor | Low-density lipoprotein receptor | P01130 | 3.7 (3.4 -- 4.1) | 3.6 (3.2 -- 4.0) |
| ITGB2 | Integrin beta-2 | P05107 | 5.4 (5.1 -- 5.7) | 5.4 (5.1 -- 5.7) |
| IL-17RA | Interleukin-17 receptor A | Q96F46 | 4.0 (3.6 -- 4.3) | 4.1 (3.8 -- 4.4) |
| TNF-R2 | Tumor necrosis factor receptor 2 | P20333 | 5.7 (5.4 -- 6.0) | 6.0 (5.7 -- 6.4) |
| MMP-9 | Matrix metalloproteinase-9 | P14780 | 5.1 (4.6 -- 5.7) | 5.2 (4.7 -- 5.8) |
| EPHB4 | Ephrin type-B receptor 4 | P54760 | 5.5 (5.2 -- 5.7) | 5.6 (5.3 -- 5.9) |
| IL2-RA | Interleukin-2 receptor subunit alpha | P01589 | 3.8 (3.5 -- 4.1) | 3.9 (3.6 -- 4.3) |
| OPG | Osteoprotegerin | O00300 | 3.9 (3.7 -- 4.2) | 4.0 (3.8 -- 4.3) |
| ALCAM | CD166 antigen | Q13740 | 7.2 (7.0 -- 7.3) | 7.2 (7.1 -- 7.4) |
| TFF3 | Trefoil factor 3 | Q07654 | 5.1 (4.8 -- 5.4) | 5.4 (5.1 -- 5.8) |
| SELP | P-selectin | P16109 | 9.3 (9.0 -- 9.7) | 9.4 (9.1 -- 9.8) |
| CSTB | Cystatin-B | P04080 | 4.0 (3.7 -- 4.4) | 4.3 (4.0 -- 4.8) |
| MCP-1 | Monocyte chemotactic protein 1 | P13500 | 4.2 (3.9 -- 4.4) | 4.2 (4.0 -- 4.5) |
| CD163 | Scavenger receptor cysteine-rich type 1 protein M130 | Q86VB7 | 7.7 (7.4 -- 8.0) | 7.8 (7.5 -- 8.1) |
| Gal-3 | Galectin-3 | P17931 | 3.2 (2.9 -- 3.4) | 3.3 (3.0 -- 3.5) |
| GRN | Granulins | P28799 | 5.4 (5.2 -- 5.6) | 5.5 (5.3 -- 5.8) |
| NT-proBNP | N-terminal prohormone brain natriuretic peptide | NA | 6.7 (5.9 -- 7.3) | 7.5 (6.6 -- 8.2) |
| BLM hydrolase | Bleomycin hydrolase | Q13867 | 2.1 (1.9 -- 2.4) | 2.2 (2.0 -- 2.5) |
| PLC | Perlecan | P98160 | 7.9 (7.7 -- 8.1) | 8.0 (7.8 -- 8.3) |
| LTBR | Lymphotoxin-beta receptor | P36941 | 3.9 (3.7 -- 4.2) | 4.1 (3.9 -- 4.4) |
| NOTCH-3 | Neurogenic locus notch homolog protein 3 | Q9UM47 | 5.7 (5.4 -- 5.9) | 5.8 (5.6 -- 6.0) |
| TIMP4 | Metalloproteinase inhibitor 4 | Q99727 | 3.6 (3.4 -- 4.0) | 3.8 (3.6 -- 4.2) |
| CNTN1 | Contactin-1 | Q12860 | 4.3 (4.1 -- 4.6) | 4.3 (4.1 -- 4.6) |
| CDH5 | Cadherin-5 | Q9NPY3 | 4.3 (4.1 -- 4.5) | 4.4 (4.2 -- 4.6) |
| TLT-2 | Trem-like transcript 2 protein | Q5T2D2 | 4.7 (4.4 -- 5.0) [2] | 4.7 (4.4 -- 5.1) [0] |
| FABP4 | Fatty acid-binding protein, adipocyte | P15090 | 5.7 (5.1 -- 6.3) | 6.0 (5.4 -- 6.9) |
| TFPI | Tissue factor pathway inhibitor | P10646 | 8.9 (8.7 -- 9.2) | 9.0 (8.8 -- 9.3) |
| PAI | Plasminogen activator inhibitor 1 | P05121 | 5.3 (4.7 -- 6.0) | 5.3 (4.6 -- 6.0) |
| CCL24 | C-C motif chemokine 24 | O00175 | 5.0 (4.4 -- 5.7) | 5.0 (4.4 -- 5.7) |
| TR | Transferrin receptor protein 1 | P02786 | 5.6 (5.1 -- 6.0) | 5.8 (5.4 -- 6.2) |
| TNFRSF10C | Tumor necrosis factor receptor superfamily member 10C | O14798 | 6.7 (6.4 -- 7.1) | 6.8 (6.4 -- 7.1) |
| GDF-15 | Growth/differentiation factor 15 | Q99988 | 6.2 (5.9 -- 6.6) | 6.6 (6.3 -- 7.1) |
| SELE | E-selectin | P16581 | 11.8 (11.3 -- 12.1) | 11.8 (11.3 -- 12.2) |
| AZU1 | Azurocidin | P20160 | 2.5 (2.1 -- 3.1) | 2.7 (2.2 -- 3.3) |
| DLK-1 | Protein delta homolog 1 | P80370 | 5.8 (5.3 -- 6.1) | 5.8 (5.4 -- 6.2) |
| SPON1 | Spondin-1 | Q9HCB6 | 2.3 (2.1 -- 2.5) | 2.4 (2.2 -- 2.7) |
| MPO | Myeloperoxidase | P05164 | 3.0 (2.8 -- 3.3) | 3.1 (2.8 -- 3.3) |
| CXCL16 | C-X-C motif chemokine 16 | Q9H2A7 | 5.2 (5.1 -- 5.4) | 5.3 (5.1 -- 5.6) |
| IL-6RA | Interleukin-6 receptor subunit alpha | P08887 | 11.6 (11.3 -- 11.9) | 11.6 (11.3 -- 11.9) |
| RETN | Resistin | Q9HD89 | 6.0 (5.7 -- 6.3) | 6.1 (5.8 -- 6.5) |
| IGFBP-1 | Insulin-like growth factor-binding protein 1 | P08833 | 4.8 (3.9 -- 5.6) | 5.3 (4.4 -- 6.0) |
| CHIT1 | Chitotriosidase-1 | Q13231 | 5.3 (4.6 -- 6.1) | 5.5 (4.8 -- 6.2) |
| TR-AP | Tartrate-resistant acid phosphatase type 5 | P13686 | 3.1 (2.9 -- 3.4) | 3.1 (2.8 -- 3.4) |
| GP6 | Platelet glycoprotein VI | Q9HCN6 | 1.9 (1.6 -- 2.3) | 2.1 (1.7 -- 2.5) |
| PSP-D | Pulmonary surfactant-associated protein D | P35247 | 3.2 (2.6 -- 3.8) | 3.4 (2.9 -- 3.9) |
| PI3 | Elafin | P19957 | 2.4 (2.0 -- 2.9) | 2.7 (2.3 -- 3.1) |
| Ep-CAM | Epithelial cell adhesion molecule | P16422 | 5.1 (4.5 -- 5.8) | 5.0 (4.4 -- 5.7) |
| AP-N | Aminopeptidase N | P15144 | 4.7 (4.5 -- 4.9) | 4.8 (4.6 -- 5.0) |
| AXL | Tyrosine-protein kinase receptor UFO | P30530 | 8.8 (8.6 -- 9.1) | 9.0 (8.7 -- 9.2) |
| IL-1RT1 | Interleukin-1 receptor type 1 | P14778 | 6.3 (6.2 -- 6.5) | 6.5 (6.3 -- 6.7) |
| MMP-2 | Matrix metalloproteinase-2 | P08253 | 3.7 (3.4 -- 3.9) | 3.8 (3.5 -- 4.1) |
| FAS | Tumor necrosis factor receptor superfamily member 6 | P25445 | 5.8 (5.6 -- 6.0) [1] | 5.8 (5.6 -- 6.1) [0] |
| MB | Myoglobin | P02144 | 7.7 (7.3 -- 8.1) | 7.8 (7.5 -- 8.3) |
| TNFSF13B | Tumor necrosis factor ligand superfamily member 13B | Q9Y275 | 6.9 (6.7 -- 7.2) | 7.1 (6.8 -- 7.4) |
| PRTN3 | Myeloblastin | P24158 | 3.7 (3.4 -- 4.1) | 3.8 (3.5 -- 4.3) |
| PCSK9 | Proprotein convertase subtilisin/kexin type 9 | Q8NBP7 | 3.1 (2.9 -- 3.4) | 3.2 (2.9 -- 3.5) |
| U-PAR | Urokinase plasminogen activator surface receptor | Q03405 | 5.5 (5.2 -- 5.7) | 5.8 (5.5 -- 6.1) |
| OPN | Osteopontin | P10451 | 7.4 (7.0 -- 7.8) | 7.7 (7.2 -- 8.1) |
| CTSD | Cathepsin D | P07339 | 2.4 (2.1 -- 2.7) | 2.5 (2.2 -- 2.9) |
| PGLYRP1 | Peptidoglycan recognition protein 1 | O75594 | 7.3 (6.9 -- 7.6) | 7.4 (7.1 -- 7.8) |
| CPA1 | Carboxypeptidase A1 | P15085 | 5.7 (5.3 -- 6.2) | 5.8 (5.3 -- 6.3) |
| JAM-A | Junctional adhesion molecule A | Q9Y624 | 3.8 (3.5 -- 4.1) | 4.1 (3.7 -- 4.4) |
| Gal-4 | Galectin-4 | P56470 | 4.1 (3.8 -- 4.5) | 4.2 (4.0 -- 4.7) |
| IL-1RT2 | Interleukin-1 receptor type 2 | P27930 | 4.9 (4.7 -- 5.1) | 4.9 (4.7 -- 5.2) |
| SHPS-1 | Tyrosine-protein phosphatase non- receptor type substrate 1 | P78324 | 3.6 (3.3 -- 3.9) | 3.7 (3.4 -- 4.0) |
| CCL15 | C-C motif chemokine 15 | Q16663 | 7.1 (6.8 -- 7.5) | 7.3 (7.0 -- 7.7) |
| CASP-3 | Caspase-3 | P42574 | 4.7 (3.9 -- 6.0) | 4.8 (4.1 -- 6.3) |
| uPA | Urokinase-type plasminogen activator | P00749 | 4.6 (4.4 -- 4.8) | 4.6 (4.4 -- 4.9) |
| CPB1 | Carboxypeptidase B | P15086 | 5.6 (5.1 -- 6.0) | 5.6 (5.2 -- 6.1) |
| CHI3L1 | Chitinase-3-like protein 1 | P36222 | 4.6 (3.9 -- 5.3) | 4.9 (4.4 -- 5.7) |
| ST2 | ST2 protein | Q01638 | 4.5 (4.1 -- 4.8) | 4.7 (4.3 -- 5.2) |
| t-PA | Tissue-type plasminogen activator | P00750 | 6.8 (6.4 -- 7.1) | 6.8 (6.4 -- 7.2) |
| SCGB3A2 | Secretoglobin family 3A member 2 | Q96PL1 | 2.7 (2.3 -- 3.2) | 2.9 (2.5 -- 3.5) |
| EGFR | Epidermal growth factor receptor | P00533 | 2.8 (2.6 -- 2.9) | 2.7 (2.6 -- 2.9) |
| IGFBP-7 | Insulin-like growth factor-binding protein 7 | Q16270 | 7.9 (7.6 -- 8.1) | 8.1 (7.9 -- 8.4) |
| CD93 | Complement component C1q receptor | Q9NPY3 | 10.9 (10.6 -- 11.1) | 11.1 (10.8 -- 11.3) |
| IL-18BP | Interleukin-18-binding protein | O95998 | 5.8 (5.6 -- 6.1) | 6.0 (5.7 -- 6.3) |
| COL1A1 | Collagen alpha-1(I) chain | P02452 | 2.7 (2.5 -- 2.9) | 2.8 (2.4 -- 3.1) |
| PON3 | Paraoxonase | Q15166 | 5.7 (5.3 -- 6.1) | 5.5 (5.1 -- 6.0) |
| CTSZ | Cathepsin Z | Q9UBR2 | 5.1 (4.9 -- 5.4) | 5.3 (5.0 -- 5.5) |
| MMP-3 | Matrix metalloproteinase-3 | P08254 | 7.7 (7.2 -- 8.1) | 7.8 (7.4 -- 8.3) |
| RARRES2 | Retinoic acid receptor responder protein 2 | Q99969 | 11.4 (11.3 -- 11.6) | 11.5 (11.3 -- 11.7) |
| ICAM-2 | Intercellular adhesion molecule 2 | P13598 | 5.4 (5.1 -- 5.6) | 5.5 (5.2 -- 5.7) |
| KLK6 | Kallikrein-6 | Q92876 | 2.4 (2.1 -- 2.6) | 2.5 (2.2 -- 2.8) |
| PDGF subunit A | Platelet-derived growth factor subunit A | P04085 | 3.1 (2.6 -- 3.8) | 3.1 (2.5 -- 3.8) |
| TNF-R1 | Tumor necrosis factor receptor 1 | P19438 | 6.7 (6.4 -- 7.0) | 7.0 (6.7 -- 7.4) |
| IGFBP-2 | Insulin-like Growth Factor-Binding Protein 2 | P18065 | 8.2 (7.6 -- 8.6) | 8.5 (8.0 -- 9.0) |
| vWF | von Willebrand factor | P04275 | 6.2 (5.7 -- 6.6) | 6.4 (5.9 -- 6.9) |
| PECAM-1 | Platelet endothelial cell adhesion molecule | P16284 | 4.1 (3.9 -- 4.3) | 4.2 (4.0 -- 4.4) |
| MEPE | Matrix extracellular phosphoglycoprotein | Q9NQ76 | 5.6 (5.3 -- 5.9) | 5.6 (5.3 -- 6.0) |
| CCL16 | C-C motif chemokine 16 | O15467 | 6.7 (6.4 -- 7.0) | 6.8 (6.5 -- 7.2) |

Continuous variables presented as median (Q1-Q3). Number of missing values presented in [n]. The CVDII panel was used for biomarkers ranging from BMP-6 to HAOX1 and CVDIII panel from TNFRSF14 to CCL16.

# **Supplemental Table 2**

Comparison between baseline characteristics of full biomarker cohort from the ARISTOTLE trial and the random sample in the identification cohort.

| Variable | Full biomarker cohort  (N = 14788) | PEA cohort  (N = 4205) |
| --- | --- | --- |
| Age (years) | 70.0 (63.0 -- 76.0) | 70.0 (63.0 -- 76.0) |
| Female | 5270 (35.6%) | 1536 (36.5%) |
| Body mass index | 28.5 (25.3 -- 32.6) [69] | 28.6 (25.4 -- 32.7) [20] |
| Current smoker | 1199 (8.1%) [14] | 373 (8.9%) [1] |
| Hypertension | 12938 (87.5%) | 3682 (87.6%) |
| Diabetes | 3659 (24.7%) | 1059 (25.2%) |
| Prior myocardial infarction | 18961 (2.8%) [1] | 546 (13.0%) [0] |
| Prior PCI/CABG | 2011 (13.6%) | 578 (13.7%) |
| Prior stroke/TIA | 2778 (18.8%) | 770 (18.3%) |
| Peripheral arterial disease | 722 (4.9%) [1] | 202 (4.8%) [0] |
| Heart failure | 4588 (31.0%) | 1309 (31.1%) |
|  |  |  |
| NT-proBNP (ng/L) | 713.0 (363.0 -- 1248.8) [10] | 700.0 (366.2 -- 1257.0) [3] |
| cTnT-hs (ng/L) | 10.9 (7.5 -- 16.7) [2] | 10.8 (7.5 -- 16.6) [0] |
| GDF-15 (ng/L) | 1383.0 (976.8 -- 2052.0) | 1373.0 (968.0 -- 2066.0) |
| Cystatin C (mg/L) | 1.0 (0.8 -- 1.2) [17] | 1.0 (0.8 -- 1.2) [4] |
| IL-6 (ng/L) | 2.3 (1.5 -- 3.9) [22] | 2.3 (1.5 -- 4.0) [1] |
| CRP | 2.2 (1.0 -- 4.8) [17] | 2.2 (1.0 -- 4.7) [4] |
| eGFR (mL/min) | 74.1 (56.7 -- 95.2) [51] | 74.5 (57.1 -- 95.8) [17] |
| GFR_MDRD | 66.4 (55.2 -- 78.6) [9] | 66.8 (55.5 -- 78.7) [1] |
| Haemoglobin (g/dL) | 14.2 (13.2 -- 15.3) [68] | 14.2 (13.1 -- 15.2) [22] |

Continuous variables presented as median (Q1-Q3). Categorical variables presented as percentage (frequency). Number of missing values presented in [n].

CABG, coronary artery bypass graft; CRP, C-reactive protein; GDF-15, growth differentiation factor 15; eGFR, estimated glomerular filtration rate; IL-6, interleukin 6; NT-proBNP, N-terminal pro-B-type natriuretic peptide; cTnT-hs, cardiac troponin T measured with high-sensitivity assay; PCI, percutaneous coronary intervention; Q, quartile; and TIA, transient ischemic attack.
